# Optimization of the antiviral spectrum of cyclophilin inhibitors targeting respiratory viruses

**DOI:** 10.1128/aac.01721-25

**Published:** 2026-07-17

**Authors:** Laurent Softic, Bryan Jimenez-Araya, Nazim Ahnou, Maxime Duval, Quentin Nevers, Rozenn Brillet, Jean-Francois Guichou, Christophe Rodriguez, Patrice Bruscella, Jean-Michel Pawlotsky, Slim Fourati, Abdelhakim Ahmed-Belkacem

**Affiliations:** 1 Team "Viruses, Hepatology, Cancer", Institut Mondor, INSERM U955, Université Paris-Est 165200 , Créteil, France; 2 Unité de Virologie et Immunologie Moléculaires (VIM), INRAE, Université Paris-Saclay 543420, Jouy-en-Josas, France; 3 Centre de Biologie Structurale (CBS), CNRS UMR 5048, INSERM U 1054, Université de Montpellier 27037, Montpellier, France; 4 Department of Virology, Hôpital Henri Mondor, AP-HP, Université Paris-Est 378967 , Créteil, France; Chinese Academy of Medical Sciences & Peking Union Medical College, Beijing, China

**Keywords:** respiratory viruses, HTA, cyclophilins, SMCypIs, broad-spectrum antivirals

## Abstract

We evaluated the antiviral activity of a library of 36 urea-based small-molecule cyclophilin inhibitors (SMCypIs) against human respiratory syncytial virus (hRSV) and human metapneumovirus (hMPV). Structure–spectrum analysis revealed virus-specific structural requirements for antiviral efficacy. By combining the key determinants identified for each virus, we rationally designed optimized dual-acting derivatives. Among these, the optimized compound F832/33 emerged as a potent, broad-spectrum antiviral agent with nanomolar activity against both hRSV and hMPV, submicromolar inhibition of cyclophilin (Cyp) peptidyl-prolyl isomerase (PPIase) activity, and no detectable cytotoxicity. Interestingly, no direct correlation was observed between Cyp PPIase inhibition and antiviral potency, suggesting that Cyp engagement rather than enzymatic inhibition *per se* is required for antiviral activity. Competition assays with alisporivir confirmed that the antiviral effects of SMCypIs depend on their specific interaction with Cyp. Beyond its dual efficacy, F832/33 displayed strong antiviral activity against additional clinically relevant respiratory pathogens, including parainfluenza virus type 3, enterovirus D68, and both ancestral and Omicron severe acute respiratory syndrome coronavirus 2 strains, in immortalized cell lines and fully differentiated human nasal epithelial cells, a physiologically relevant model of respiratory infection. Collectively, our results demonstrate that structure–activity relationship-guided optimization can be successfully applied to expand the antiviral spectrum of SMCypIs, positioning F832/33 as a promising host-targeted broad-spectrum antiviral candidate. Further *in vivo* studies are warranted to evaluate its therapeutic potential and clarify its precise mechanism of action.

## INTRODUCTION

Acute respiratory viral infections are a leading cause of morbidity and mortality in children under 5 years of age. These infections pose a significant health risk to immunocompromised patients and the elderly, especially in low- and middle-income countries where access to healthcare is limited (1, 2). Acute respiratory viral infections primarily target the lower respiratory tract and are characterized by symptoms including cough, nasal congestion, and mild fever. These symptoms can progress to severe complications, including pneumonia and acute respiratory distress syndrome (3, 4).

The increasing threat of viral respiratory outbreaks driven by climate change, urbanization, population aging, and sociological shifts challenges global preparedness. These factors contribute to seasonal epidemics and the emergence of zoonotic spillovers, new viral variants, and pathogens with pandemic potential (5, 6). Multiple respiratory viruses co-circulate annually in the global population. Since the 2022–2023 season, most regions have experienced a concurrent surge in human respiratory syncytial virus (hRSV), severe acute respiratory syndrome coronavirus 2 (SARS-CoV-2), and influenza viruses, collectively referred to as the “tripledemic.” This unprecedented co-circulation has resulted in millions of infections and considerable mortality, placing a significant strain on the healthcare systems worldwide (7, 8). Additionally, there is increasing evidence highlighting the previously underestimated burden of human metapneumovirus (hMPV), particularly among vulnerable populations (9, 10).

In response, multiple preventive strategies have been developed, including active and passive immunoprophylaxis against influenza, hRSV, and SARS-CoV-2, as well as a vaccine for hMPV that is currently under development (11). Despite these advances, therapeutic options remain limited, as there is still no approved antiviral for two of the most clinically relevant respiratory viruses: hRSV and hMPV. The SARS-CoV-2 pandemic underscored the importance of pandemic preparedness and the urgent need to develop effective, broad-spectrum antiviral strategies (12). Developing virus-specific, direct-acting antivirals for each existing and emerging respiratory virus is unlikely to be feasible, given the time and costs involved. Thus, after decades of effort, the current antiviral arsenal remains sparse (13). In this context, host-targeted, broad-spectrum antivirals represent a promising complementary approach to direct-acting antivirals. They offer the potential to counter both seasonal epidemics and future pandemics (13, 14).

Cyclophilins (Cyps) are a family of peptidyl-prolyl *cis-trans* isomerases (PPIase) that are involved in protein folding. Cyps play key roles in the life cycles of various viruses, including the hepatitis C virus (HCV) (15), the human immunodeficiency virus (HIV) (16), several coronaviruses (17), and the influenza A virus (18), making Cyps an interesting target for developing broad-spectrum antiviral drugs (19). Using a fragment-based drug discovery approach, we previously generated a family of non-peptidic, urea-based, small-molecule cyclophilin inhibitors (SMCypIs). SMCypIs are modular, with three variable moieties: R1 interacts with the catalytic pocket, R3 binds to the gatekeeper pocket, and R2 is positioned at the interface of the two binding sites (20, 21). SMCypIs have demonstrated potent antiviral activity against HIV, human coronavirus 229E (hCoV-229E), HCV, and several flaviviruses (20, 22)

In this study, we evaluated the antiviral activity of several SMCypIs against two major respiratory pathogens, hRSV and hMPV, for which no antiviral therapies are currently available. We identified virus-specific structural determinants of the antiviral effect and combined them to generate optimized derivatives with dual antiviral efficacy. We assessed the activity spectrum of the lead compound, F832/33, against other clinically relevant respiratory viruses, including parainfluenza virus type 3 (PIV-3), enterovirus D68 (EV-D68), and ancestral and an Omicron strain of SARS-CoV-2. Finally, we validated the broad-spectrum antiviral activity of F832/33 in primary human nasal epithelial cells (hNECs), which are a physiologically relevant *ex vivo* model for respiratory viral diseases. Our findings highlight the potential of structure–activity relationship (SAR)-guided optimization to broaden the antiviral spectrum of SMCypIs. These results establish SMCypIs as promising, adaptable tools for combating current and future respiratory virus outbreaks. Further investigations are warranted to confirm the *in vivo* efficacy of SMCypIs as host-targeted broad-spectrum antivirals and to elucidate their precise mechanism of action.

## MATERIALS AND METHODS

### Cells

African green monkey kidney epithelial cells (Vero, ATCC CCL-81), Vero-TMPRSS2 cells, human larynx epidermoid carcinoma cells (HEp-2, ATCC CCL-23), and human lung carcinoma epithelial cells (A549, ATCC CCL-185) were cultured in complete Dulbecco’s Modified Eagle Medium (DMEM, ThermoFisher Scientific, Waltham, Massachusetts) supplemented with 10% fetal bovine serum, 50 international units (IU)/mL penicillin, 100 µg/mL streptomycin, and 0.1 µg/mL amphotericin B (ThermoFisher Scientific).

Human nasal epithelial cells (MucilAir Pool Nasal) were ordered from Epithelix (Geneva, Switzerland) and cultured at the air-liquid interface. MucilAir medium was added to the lower chamber of the wells and changed every 2–3 days.

### Viruses

hRSV-A (ATCC VR-26), PIV-3 (ATCC VR-93), and EV-D68 (ATCC VR-1826) were ordered from the American Type Culture Collection (ATCC). hMPV (strain: NL/1/00; genotype: A1) was obtained from the European Virus Archive (EVAg, ref SKU: 011V-00930). The SARS-CoV-2 ancestral strain B.117.18 was isolated from nasopharyngeal swabs collected at Henri Mondor Hospital. The Omicron B.1.1.529 variant was ordered from EVAg (ref SKU: 001V-04653).

### Compound synthesis

Compound synthesis was performed by AGV Discovery (Montferrier-sur-Lez, France) as described in our previous reports (20–22). The nuclear magnetic resonance (NMR) characterization of compounds F832, F833, F836, F832/33, and F832/36 is provided in the Supplemental material.

### PPIase enzyme assay

CypA and CypD PPIase activities were measured at 20°C using the standard chymotrypsin-coupled assay. The assay buffer (25 mM HEPES and 100 mM NaCl, pH 7.8) and the purified CypA and CypD (expressed and purified in-house as described in reference 20, 1,900 nM stock solution) were precooled to 4°C. Then, 5 mL of 50 mg/mL chymotrypsin in 1 mM HCl was added. The reaction was initiated by adding 20 mL of a 3.2 mM peptide substrate (Suc-Ala-Ala-Cis-Pro-Phe-pNA) in a LiCl/TFE solution with rapid inversion. After a brief delay following the onset of mixing, the absorbance of p-nitroaniline was monitored at 390 nm until the reaction was complete (1 min). The final concentration of LiCl in the assay was 20 mM, and tetrafluoroethylene (TFE) was present at a concentration of 4% (vol/vol). Absorbance readings were collected every second using a spectrophotometer. To assess inhibition, increasing concentrations of the tested compound, dissolved in dimethyl sulfoxide, were added to the Cyp solution in the assay buffer. The percent inhibition of Cyp PPIase activity was calculated from the slopes, and the obtained values represent the mean ± standard deviation (SD) of at least two independent measurements.

### Cellular viability

Cellular viability in immortalized cells (A549, Vero, and HEp-2 cells) was assessed using the CytoTox-Glo Cytotoxicity Assay (Promega), according to the manufacturer’s recommended procedure.

### Viral RNA extraction and RT-qPCR

Total RNA was extracted from infected cells and cell culture supernatant using the RNeasy Mini Kit (Qiagen, Hilden, Germany) and the QIAamp Viral RNA Mini Kit (Qiagen), respectively. Viral RNA was quantified using the GoTaq Probe One-Step RT-qPCR System (Promega, Madison, Wisconsin) with specific primers. RT-qPCR was performed using a QuantStudio 5 Real-Time PCR System (ThermoFisher Scientific). Viral RNA levels were quantified using the ΔΔCT method and normalized to GAPDH mRNA or 18S rRNA for intracellular samples.

### Immunoblotting

The cells were lysed using a buffer solution containing 20 mM Tris-HCl, 100 mM NaCl, 1 mM EDTA, 0.5% NP-40, and 10% glycerol. The cell lysates were then incubated at 95°C for 5 min with XT Sample Buffer and XT Reducing Agent (Bio-Rad, Hercules, California). The samples were then loaded onto a 4%–12% polyacrylamide gel (Bio-Rad) and transferred to a nitrocellulose membrane (ThermoFisher Scientific) for electrophoresis. Viral protein detection was performed using primary antibodies specific to the following: hRSV M2-1 protein (Abcam, ab94805), PIV-3 HN protein (Abcam, ab49756), hMPV (Abcam, ab94802), EV-D68 VP1 (GeneTex, GTX633770), and SARS-CoV-2 N (GeneTex, GTX135357). Protein size was determined using the Precision Plus Protein Dual Color Standards weight marker (Bio-Rad).

Protein bands were detected using an Amersham ECL Prime Western blotting detection reagent (Merck, Rahway, New Jersey) and visualized with an ImageQuant LAS 4000 mini (GE Healthcare, Chicago, Illinois).

### Transepithelial electrical resistance measurement

Epithelial integrity of hNECs was evaluated using an EVOM volt-ohmmeter (World Precision Instruments, Sarasota, Florida) to measure transepithelial electrical resistance (TEER).

### Statistical analysis

Statistical analyses were performed using GraphPad Prism, version 10.1.0. Data are presented as the mean ± SD. Comparisons between two groups were performed using a two-tailed Student’s *t*-test. Statistical significance was defined as follows: *P < 0.05; **P < 0.01.

## RESULTS

### Screening of SMCypIs against hMPV and hRSV RNA production

**Fig 1:**
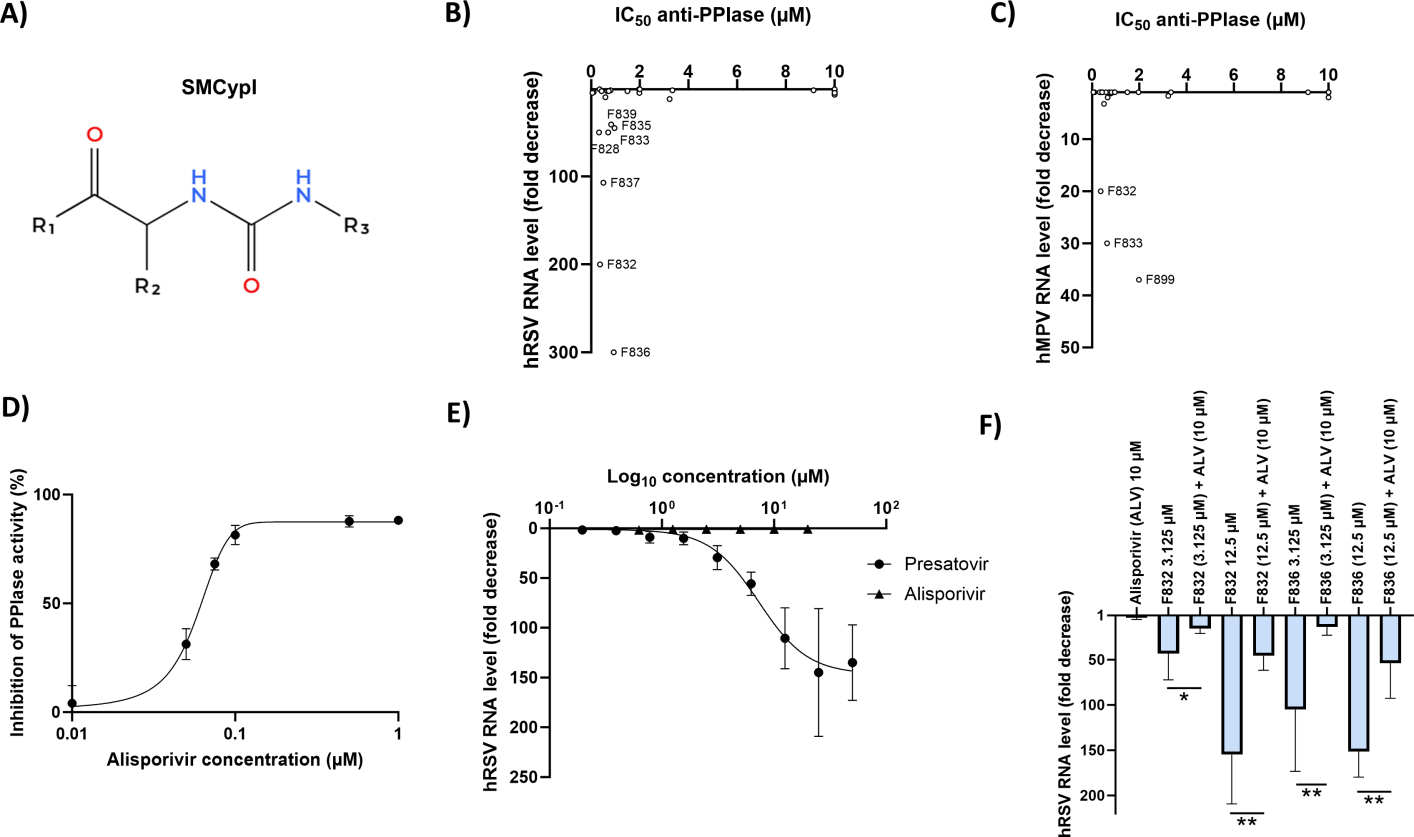
(**A**) General chemical structure of the SMCypIs with three variable moieties: R1, R2, and R3. (**B**) Relationship between antiviral activity against hRSV of 36 SMCypIs and their PPIase inhibitory activity, as measured by the IC_50_ of enzyme activity with purified CypA. (**C**) Relationship between antiviral activity against hMPV of 36 SMCypIs and their PPIase inhibitory activity, as measured by the IC_50_ of enzyme activity with purified CypA. (**D**) Dose-response inhibition of CypA PPIase activity by alisporivir. (**E**) Antiviral activity of presatovir and alisporivir against hRSV. (**F**) Antiviral activity of compounds F832 and F836 against hRSV at 3.125 and 12.5 µM, alone and in competition with alisporivir at 10 µM in HEp-2 cells. The data are presented as the mean ± standard deviation (SD) from at least two independent experiments. A two-tailed Student’s *t*-test was applied. * *P*-value < 0.05; ** *P*-value < 0.01.

Taking advantage of the structural plasticity and ease of synthesis of urea-based SMCypIs (Fig. 1A), we synthesized a library of 36 derivatives with different R1, R2, and R3 substituents. These compounds were evaluated for antiviral activity at a concentration of 25 µM against hRSV and hMPV by quantifying viral RNA levels using RT-qPCR in appropriate cell lines.

Of the 36 compounds tested, 10 displayed significant antiviral activity against hRSV, which is defined as a reduction of more than 1 log_10_ in intracellular viral RNA (Table 1). Seven compounds reduced hRSV RNA levels by 1–2 log_10_, and three achieved reductions greater than 2 log_10_. In contrast, only three compounds reduced hMPV viral RNA by more than 1 log_10_, suggesting that SMCypIs have a stronger antiviral effect against hRSV (Table 1).

**TABLE 1:** Chemical structure, antiviral activity (hRSV and hMPV), and CypA PPIase inhibition potency of SMCypIs

Compound name	R1	R2	R3	hRSV RNA level (fold decrease)	hHMPV RNA level (fold decrease)	IC_50_ CypA PPIase activity (µM)
F671	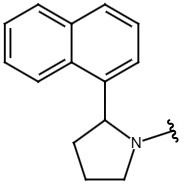		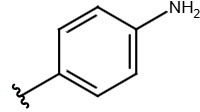	3	1	1.49 ± 0.45
F672	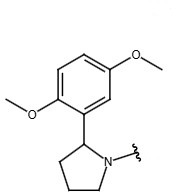		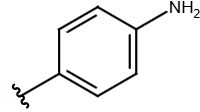	2	1	9.14 ± 8.62
F681	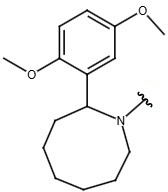		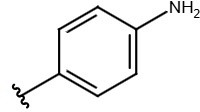	2	1	>10
F682	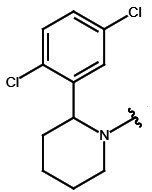		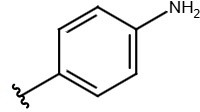	1	1	ND
F686	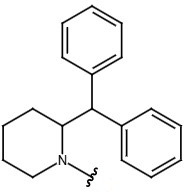		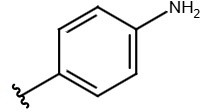	1	1	ND
F712	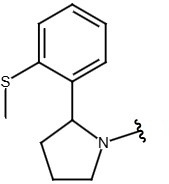	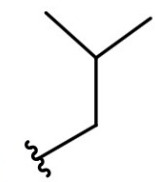	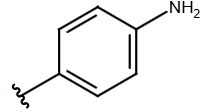	2	1	3.34 ± 1.42
F716	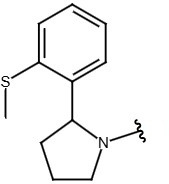	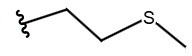	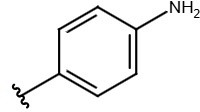	2	1	0.79 ± 0.12
F728	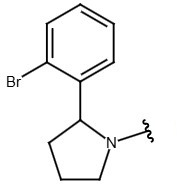		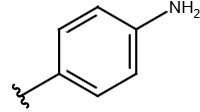	1	0.6	1.97 ± 0.42
F759	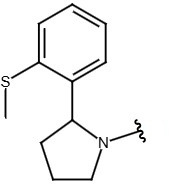	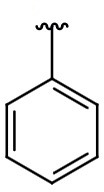	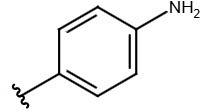	4	1	0.1 ± 0.01
F766	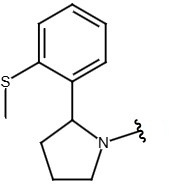	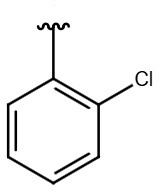	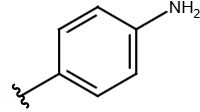	3	1	0.42 ± 0.10
F767	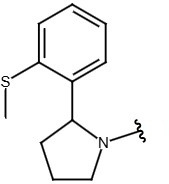	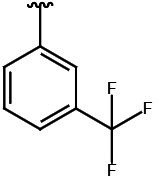	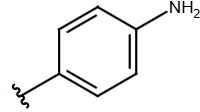	12	2	3.22 ± 0.95
F768	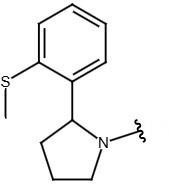	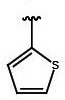	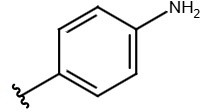	3	1	ND
F798	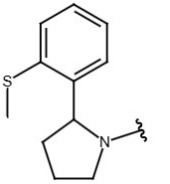	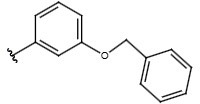	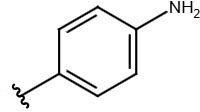	1	1	0.34 ± 0.11
F799	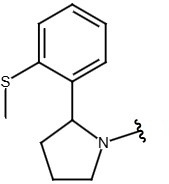	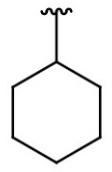	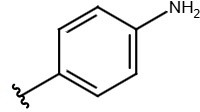	3	2	0.66 ± 0.12
F800	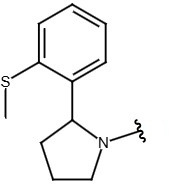	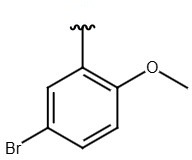	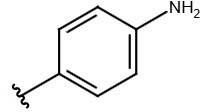	5	1	0.05 ± 0.01
F816	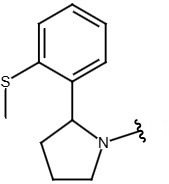	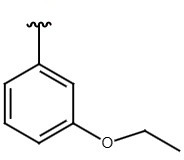	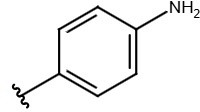	3	1	ND
F819	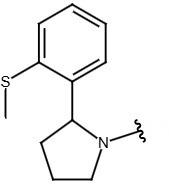	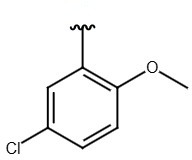	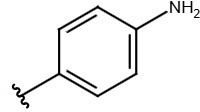	10	1	0.59 ± 0.18
F823	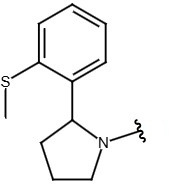	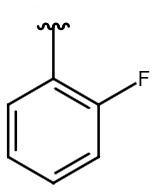	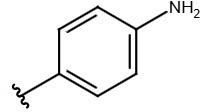	5	1	ND
F824	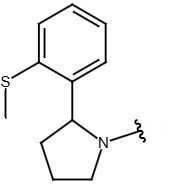	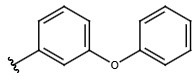	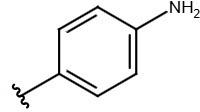	1	1	ND
F826	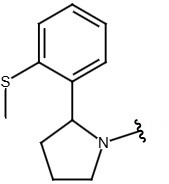	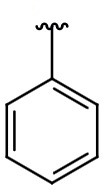	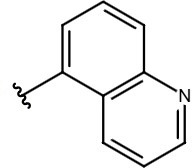	4	1	ND
F827	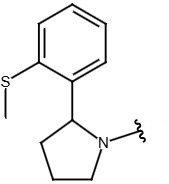	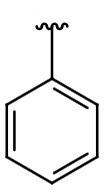	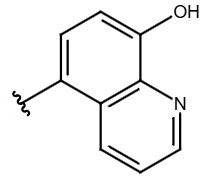	5	2	>10
F828	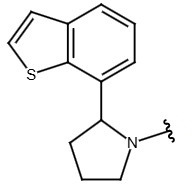	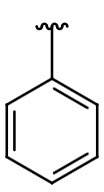	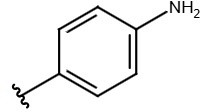	50	1	0.32 ± 0.02
F831	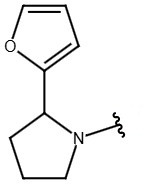	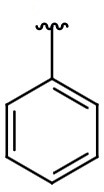	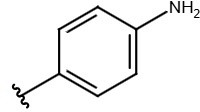	2	1	>10
F832	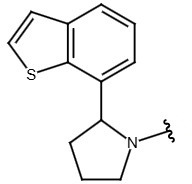	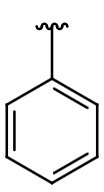	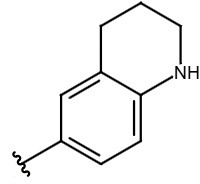	200	20	0.36 ± 0.09
F833	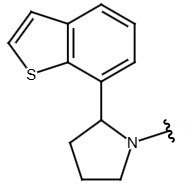	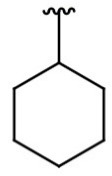	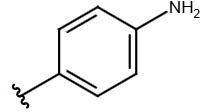	50	30	0.63 ± 0.6
F835	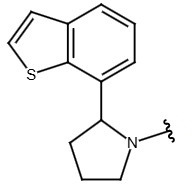	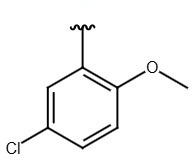	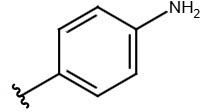	45	1	0.96 ± 0.9
F836	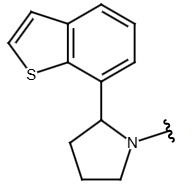	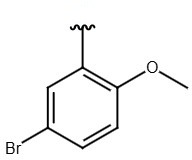	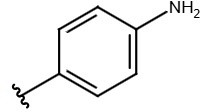	300	1	0.93 ± 0.04
F837	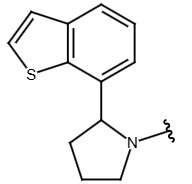	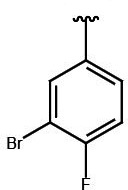	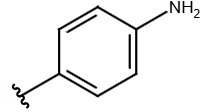	107	3	0.50 ± 0.40
F839	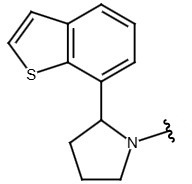	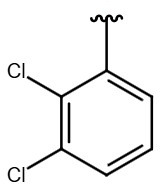	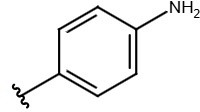	41	1	0.82 ± 0.09
F840	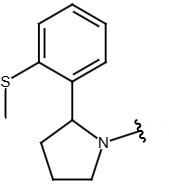	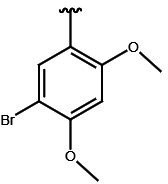	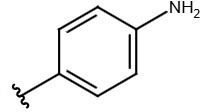	10	1	0.74 ± 0.05
F843	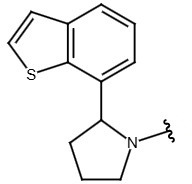	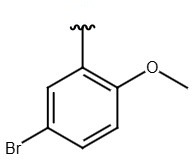	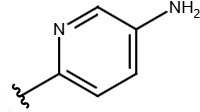	7	1	>10
F849	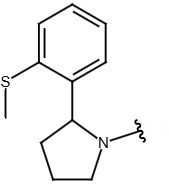	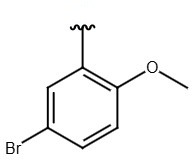	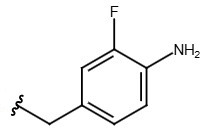	3	1	0.72 ± 0.07
F857	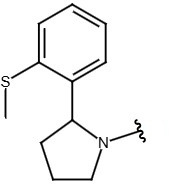	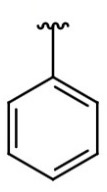	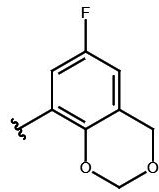	5	1	>10
F868	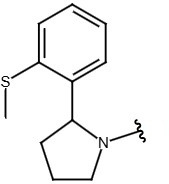	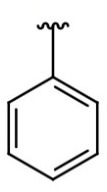	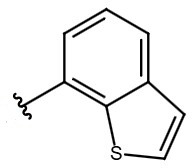	5	1	>10
F870	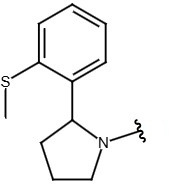	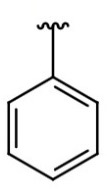	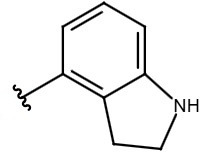	7	1	>10
F899	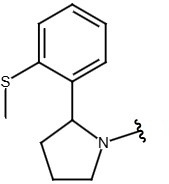	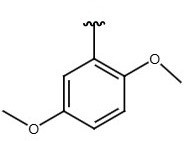	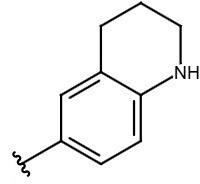	5	37	1.98 ± 0.52
Alisporivir	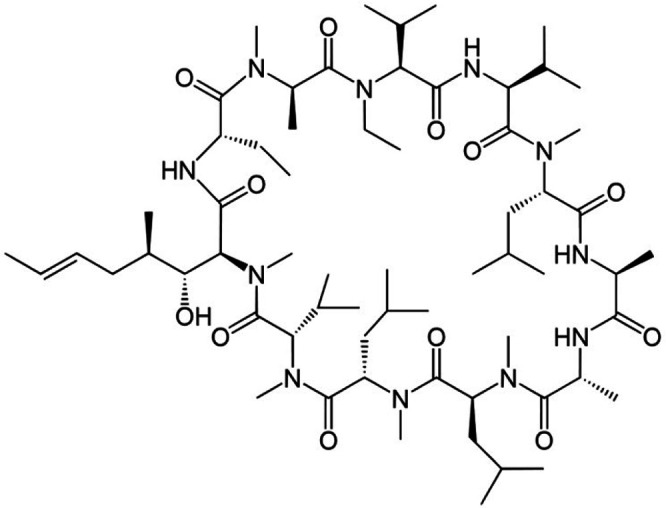			1	1	0.05 ± 0.01

### Relationship between PPIase inhibition and the antiviral activity of SMCypIs

The inhibitory potency of the 36 SMCypIs against CypA PPIase activity was determined *in vitro* using a purified enzyme (Table 1). The IC₅₀ values for anti-PPIase activity were plotted against anti-hRSV activity (Fig. 1B) and anti-hMPV activity (Fig. 1C).

Interestingly, no correlation was observed between anti-PPIase activity and antiviral efficacy for either virus. While all seven anti-hRSV active compounds had submicromolar PPIase IC₅₀ values, nine other derivatives with similar or greater anti-PPIase potency did not inhibit hRSV replication. A similar trend was observed for hMPV: 14 submicromolar PPIase inhibitors showed no detectable antiviral activity. These findings suggest that inhibiting PPIase is insufficient to confer antiviral efficacy. Therefore, other cyclophilin-dependent or -independent mechanisms likely contribute to viral restriction.

To confirm this dissociation, we evaluated the ability of alisporivir, a macrocyclic cyclophilin inhibitor, to inhibit CypA PPIase activity (Fig. 1D) and hRSV RNA production (Fig. 1E). As expected, alisporivir potently inhibited CypA enzymatic activity. However, it failed to reduce hRSV RNA levels. In contrast, presatovir, a well-characterized hRSV fusion inhibitor, efficiently reduced viral replication (Fig. 1E).

The intriguing differential effect between SMCypIs and alisporivir prompted us to further investigate whether SMCypI antiviral activity is specifically related to Cyp binding. Cyclosporin A and alisporivir, a non-immunosuppressive analog, are known to compete with SMCypIs for Cyp binding, although they adopt distinct binding modes (20, 22). To address this, we performed an *in cellulo* competition assay in HEp-2 cells infected with hRSV. We tested the effect of adding alisporivir on two representative SMCypIs: F832 and F836 (Fig. 1F). We observed that alisporivir significantly reduced the anti-hRSV potency of both compounds, which is consistent with a competitive interaction at the level of Cyp binding.

### Structure–activity relationship analysis

SAR analysis revealed virus-specific chemical determinants of SMCypI antiviral activity (Table 1). The presence of a benzothiophene at position R1 was strongly associated with anti-hRSV potency. For example, compound F828, which harbors a benzothiophene at R1, is substantially more effective than its analog, F759, which has a thioanisole at the same position. In contrast, the presence of a benzothiophene at position R1 was not critical for anti-hMPV activity. In fact, the most potent anti-hMPV SMCypI is F899, which has a thioanisole group at R1 (Table 1).

The presence of phenyl or cyclohexyl groups at position R2 was favorable for SMCypI antiviral activity against both viruses, suggesting that these groups play a role in the SMCypIs’ broad-spectrum potency. Halogen substitution on the phenyl ring of R2 enhanced anti-hRSV potency (e.g., F828 vs F836), but did not improve anti-hMPV activity (Table 1).

The R3 position also played a role in virus-specific antiviral effects. SMCypIs with anti-hRSV activity had an aniline at R3, except for F832. Tetrahydroquinoline, on the other hand, was more common among derivatives active against hMPV (Table 1).

**Fig 2:**
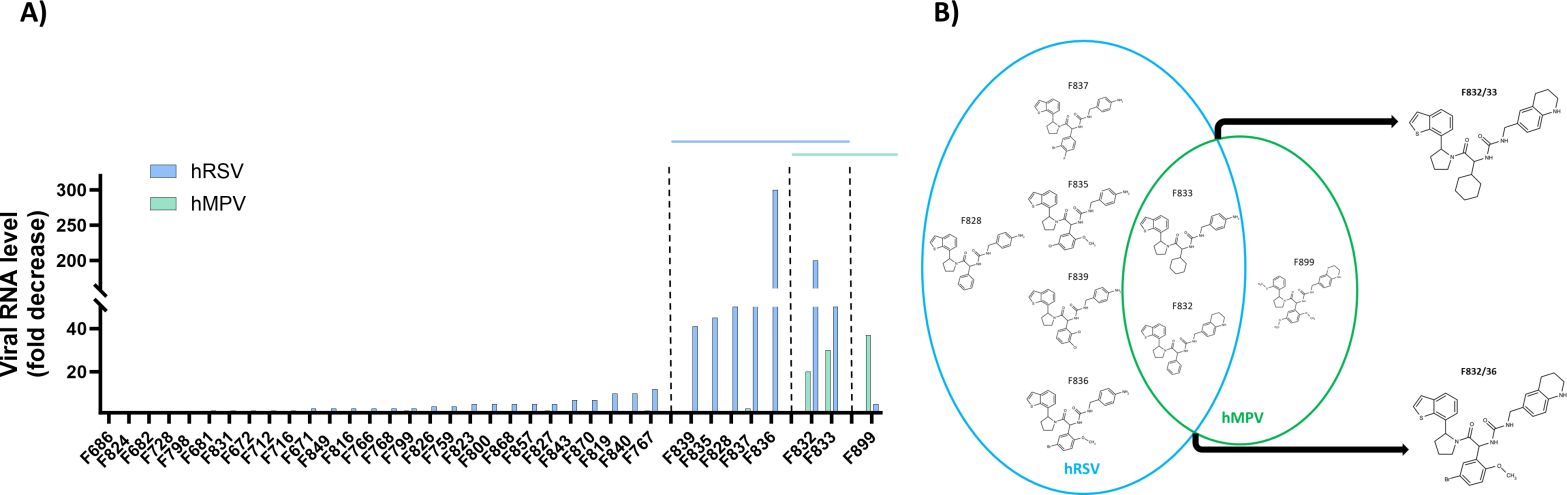
(**A**) Cross-spectrum analysis of the anti-hRSV (blue) and anti-hMPV (green) activities of 36 SMCypIs. Compounds F832 and F833 inhibited both viruses. (**B**) Chemical structures of the seven SMCypIs effective against hRSV and the three SMCypIs effective against hMPV revealing virus-specific determinants of activity. This provides the basis for designing the two hybrid molecules, F832/33 and F832/36.

Cross-spectrum analysis revealed that only two compounds, F832 and F833, inhibited both hRSV and hMPV (Fig. 2A and B). Structure–activity relationship revealed that the presence of a benzothiophene at position R1 was critical for anti-hRSV activity, while the presence of a tetrahydroquinoline at position R3 favored anti-hMPV activity. These insights provided the basis for the design of optimized, dual-acting compounds.

### Design and characterization of optimized, dual-acting SMCypI derivatives

To optimize both antiviral potency and spectrum breadth, two hybrid molecules were designed and synthesized (Fig. 2B). F832/33 was generated by combining the R1 and R3 moieties of F832 with the R2 group of F833; these are both dual-active scaffolds that were previously identified. F832/36 was derived from F832 and F836 by incorporating the R1 and R2 moieties of F836 and the R3 moiety of F832.

**Fig 3:**
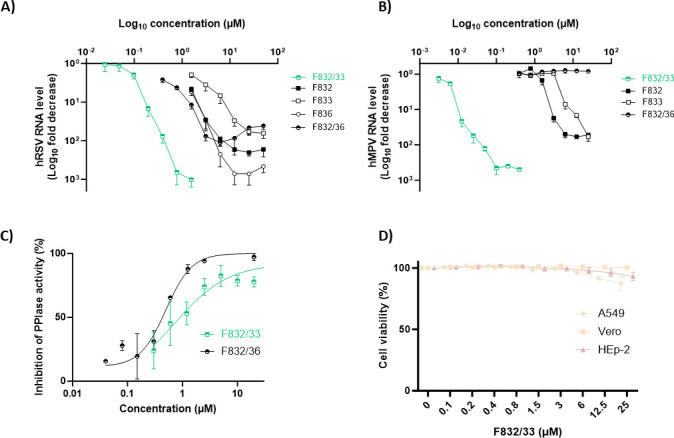
*In vitro* activity of parental compounds F832, F833, and F836, as well as optimized derivatives F832/33 (green) and F832/36, against hRSV (**A**) and hMPV (**B**). (**C**) Inhibition of CypA PPIase activity by F832, F833, and their derivative F832/33. (**D**) Cell viability assay in A549, Vero, and HEp-2 immortalized cell lines exposed to increasing concentrations of F832/33. Data are shown as mean ± SD from at least two independent experiments.

We evaluated the dose-dependent activity of F832/33, F832/36, and their parent compounds against hRSV and hMPV. F832/36 exhibited slightly increased anti-hRSV activity compared to its parent compounds (Fig. 3A; Table 2). However, F832/36 did not display significant anti-hMPV activity, which is consistent with the lack of efficacy of its parent compound, F836 (Fig. 3B; Table 2). Substituting the aniline group (R3) with tetrahydroquinoline was insufficient to restore hMPV activity. Furthermore, halogen substitution at R2 further impaired potency. Due to its limited dual antiviral spectrum, F832/36 was not pursued further.

**TABLE 2:** CypA and CypD PPIase inhibition potency and antiviral activity (hRSV and hMPV) of SMCypI parent compounds and optimized derivatives

	IC_50_ of cyclophilin A PPIase activity (µM)	IC_50_ of cyclophilin D PPIase activity (µM)	EC_50_ of hRSV RNA production	EC_50_ of hMPV RNA production
F832	0.36 ± 0.09	0.22 ± 0.06	5.70 ± 1.81	3.87 ± 0.91
F833	0.63 ± 0.6	0.17 ± 0.05	26.90 ± 6.98	25.10 ± 6.02
F836	0.93 ± 0.04	0.1 ± 0.02	8.45 ± 2.98	>25
F832/33	0.73 ± 0.54	1.26 ± 0.11	0.81 ± 0.08	0.07 ± 0.04
F832/36	0.49 ± 0.15	0.35 ± 0.16	1.81 ± 0.15	>25

In contrast, F832/33 retained and significantly improved antiviral activity against both viruses. It exhibited EC₅₀ values of 800 nM and 70 nM against hRSV and hMPV, respectively (Fig. 3A and B; Table 2), making it substantially more potent than its parent scaffolds.

The inhibitory potency of F832/33 and F832/36 against CypA was assessed *in vitro* (Fig. 3C). Both compounds inhibited PPIase activity with IC₅₀ of 0.73 ± 0.54 µM (F832/33) and 0.49 ± 0.15 µM (F832/36). Although the design did not enhance enzymatic inhibition compared to parent compounds, F832/33 exhibited markedly improved antiviral potency. Similar results were observed when assessing CypD PPIase inhibition (Table 2). These results confirm that antiviral activity is not solely dependent on anti-PPIase activity. Consequently, cytotoxicity of F832/33 was assessed in three different cell lines. As shown in Fig. 3D, F832/33 did not significantly reduce cell viability at concentrations up to 25 µM, which indicates a favorable selectivity index.

Taken together, these results demonstrate the feasibility of tailoring SMCypIs for enhanced antiviral breadth and potency. F832/33, identified through rational SAR-guided design, emerged as a promising lead compound with potent dual activity against both hRSV and hMPV, robust anti-CypA activity, and low cytotoxicity. Consequently, F832/33 was selected for further exploration of its expanded antiviral spectrum

### F832/33 antiviral activity against respiratory viruses

**Fig 4:**
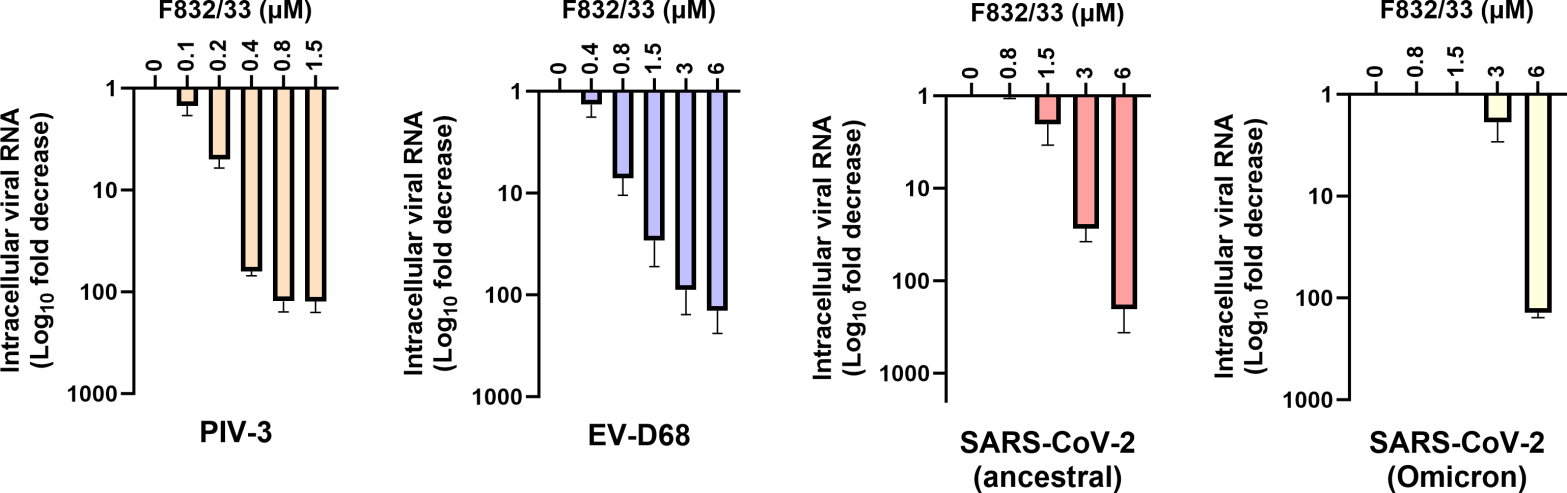
Reduction of intracellular viral RNA levels of four respiratory viruses (PIV-3, EV-D68, SARS-CoV-2 ancestral strain, and SARS-CoV-2 Omicron variant) by increasing concentrations of F832/33 in appropriate immortalized cell lines (PIV-3 and ancestral SARS-CoV-2: Vero cells; EV-D68: A549 cells; SARS-CoV-2 Omicron: VeroTMPRSS2 cells). Data are shown as the mean ± SD from at least two independent experiments.

The antiviral efficacy of F832/33 against PIV-3, EV-D68, and both the ancestral and Omicron strains of SARS-CoV-2 was evaluated in their respective permissive cell lines, as described in the Materials and Methods section. As shown in Fig. 4, F832/33 consistently inhibited the replication of all four viruses in a dose-dependent manner. At the highest tested concentration, F832/33 reduced intracellular viral RNA levels by 124-fold for PIV-3, 143-fold for EV-D68, 200-fold for the ancestral SARS-CoV-2 strain, and 140-fold for the Omicron variant.

**Fig 5:**
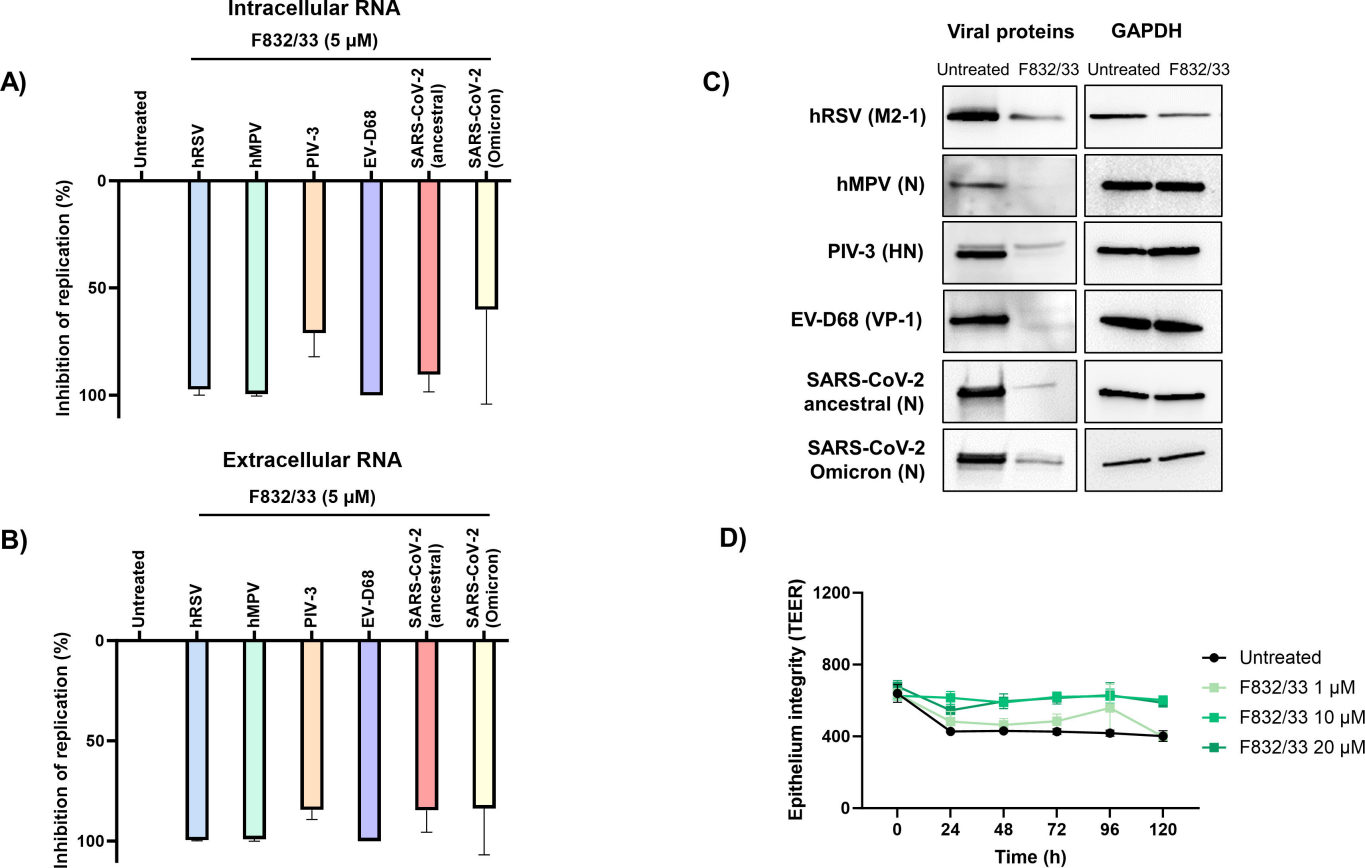
Antiviral activity of F832/33 against six respiratory viruses (hRSV, hMPV, PIV-3, EV-D68, SARS-CoV-2 ancestral strain, and SARS-CoV-2 Omicron variant) in hNECs. (**A**) Inhibition of intracellular production of viral RNA by F832/33 at a concentration of 5 µM, 72 h post-infection. Data are shown as the mean ± SD from at least two independent experiments. (**B**) Inhibition of extracellular production of viral RNA by F832/33 at a concentration of 5 µM, 72 h post-infection. Data are shown as the mean ± SD from at least two independent experiments. (**C**) Inhibition of viral protein production by F832/33 at a concentration of 5 µM, 72 h post-infection measured by western blot using the following antibodies: hRSV: M2-1; PIV-3: HN; hMPV: hMPV protein; EV-D68: VP1; SARS-CoV-2: N. The images are representative of at least two independent experiments. (**D**) Transepithelial electrical resistance (TEER) measurements assessing epithelial integrity after hNEC exposure to F832/33.

To further validate these findings in a highly physiologically relevant model, the antiviral activity of F832/33 was assessed in a well-differentiated primary hNEC model, which closely mimics the natural site of infection of respiratory viruses. hNEC were infected with six representative viruses (hRSV, hMPV, PIV-3, EV-D68, and the ancestral and Omicron SARS-CoV-2 strains) and treated with 5 µM of F832/33. Viral RNA replication was measured 72 h post-infection by quantifying both intracellular and extracellular viral RNA, as well as viral protein expression (Fig. 5).

F832/33 markedly decreased intracellular viral RNA levels for all six viruses tested, reducing them by 97% for hRSV, 99% for hMPV, 71% for PIV-3, 99% for EV-D68, 90% for the ancestral SARS-CoV-2 strain, and 60% for the SARS-CoV-2 Omicron variant (Fig. 5A). Extracellular viral RNA production was also strongly reduced, with inhibition reaching 99% for hRSV and hMPV, 84% for PIV-3, 99% for EV-D68, 85% for the ancestral SARS-CoV-2 strain, and 84% for the Omicron variant of SARS-CoV-2 (Fig. 5B).

Western blot analysis confirmed the potent suppression of viral protein expression across all six viruses with F832/33 treatment (Fig. 5C). F832/33 did not alter epithelial integrity at the tested concentration, as demonstrated by stable TEER values in treated hNECs (Fig. 5D).

Taken together, these results establish F832/33 as a broad-spectrum and potent antiviral agent effective against multiple clinically relevant respiratory viruses, including paramyxoviruses, enteroviruses, and coronaviruses, in both immortalized cell lines and a physiologically relevant primary epithelial model.

## DISCUSSION

The development of antiviral therapies has transformed the treatment of chronic viral infections, including those related to HIV, hepatitis B virus, and HCV. Currently approved drugs are mostly direct-acting antivirals (DAAs), which target a specific viral protein function essential to the virus’s life cycle. This limits their spectrum of action to their target virus. In addition, developing them for other viral infections requires high development costs and long timelines (23, 24). Broad-spectrum, host-targeted antivirals offer a valuable alternative to DAAs. They block a cellular function that plays a key role in the life cycle of a virus, family of viruses, or viruses from different families that require this cellular function. Thus, host-targeted antiviral approaches combine faster, cost-effective development with the ability to prepare for emerging infections, cover rare infections, and be used before a precise etiological diagnosis is made, increasing the likelihood of timely viral control.

A decade ago, we developed and characterized a novel family of SMCypIs that exhibit potent antiviral activity against HIV, the human coronavirus 229E (hCoV-229E), HCV, and several flaviviruses (20, 22). In this study, we synthesized and characterized a new SMCypI, F832/33, which exhibits potent dual activity against hRSV and hMPV. It also has extended efficacy against a variety of clinically relevant respiratory viruses, including PIV-3, EV-D68, and different SARS-CoV-2 strains. Using a structure–activity relationship-guided approach, we demonstrated that subtle modifications to our urea-based SMCypIs enabled the fine-tuning of their antiviral spectra. Structure-spectrum analysis revealed virus-specific requirements for activity. Specifically, a benzothiophene at position R1 and a halogen-substituted phenyl group at position R2 enhanced anti-hRSV activity, and the presence of a tetrahydroquinoline at position R3 correlated with anti-hMPV activity. Combining these determinants in a single molecule resulted in F832/33, which exhibited substantially enhanced dual activity compared to its parent scaffolds, F832 and F833. These results demonstrate the feasibility of rationally designing host-targeted compounds to expand their antiviral spectrum. They also highlight the adaptability of the SMCypI scaffold as a flexible chemical platform.

A major finding of this study is the lack of correlation between inhibiting cyclophilin PPIase activity and the antiviral potency of SMCypIs. While most SMCypIs with antiviral properties exhibited submicromolar IC₅₀ values against CypA PPIase activity, several potent PPIase inhibitors were ineffective against viral replication. Furthermore, alisporivir, a potent macrocyclic inhibitor of CypA PPIase activity, did not affect hRSV replication. Thus, enzymatic inhibition alone cannot explain antiviral efficacy, suggesting alternative, cyclophilin-dependent or -independent mechanisms.

Competition experiments using alisporivir and SMCypIs (F832 and F836) demonstrated that the antiviral effect of SMCypIs requires Cyp engagement, but likely through interactions distinct from those involved in classical macrocyclic inhibitors. Structural analyses support this notion: unlike alisporivir, SMCypIs interact with both the catalytic pocket and an adjacent “gatekeeper” pocket, potentially modulating cyclophilin–protein interactions (20). Binding of SMCypIs could disrupt Cyp interactions that play a key role in viral life cycles independent of PPIase inhibition. This finding is consistent with recent studies highlighting the non-enzymatic roles of Cyps in viral replication (25–27). A unifying mechanism explaining the role of Cyps in viral life cycles has yet to be established. Cyp proteins are increasingly recognized as modulators of protein–protein interactions and complex stabilization, regardless of their PPIase activity. Their multifunctionality, along with our results, establishes Cyps as a promising foundation for developing antiviral molecules that modulate protein–protein interactions, including “molecular glue” (28) and PROTAC strategies (29). Beyond their therapeutic potential, SMCypIs are powerful chemobiological tools for revealing non-canonical Cyp roles in viral life cycles. This is exemplified by the newly discovered Cyp dependencies of PIV-3, hMPV, and EV-D68 described in this study, as well as by the confirmation of the involvement of Cyps in the replication cycles of SARS-CoV-2 and hRSV, as previously reported (27, 30, 31).

As shown here, F832/33 inhibited the replication of various respiratory viruses belonging to three major families (Paramyxoviridae, Picornaviridae, and Coronaviridae) in immortalized cell lines and in the highly physiologically relevant model of hNECs, where it markedly reduced viral RNA levels, protein expression, and extracellular release, without compromising the integrity of the epithelial barrier. Our study provides strong preclinical evidence of the antiviral potential of F832/33. Furthermore, F832/33 has recently demonstrated efficacy against feline and porcine alpha-coronaviruses, highlighting its potential to mitigate zoonotic viral emergence (32). However, several questions remain. The molecular interactions disrupted by SMCypIs during viral replication have yet to be identified. Future proteomic and structural investigations are essential for mapping cyclophilin-protein complexes involved in the life cycles of hRSV, hMPV, PIV-3, EV-68, and SARS-CoV-2. *In vivo* efficacy and pharmacokinetic profiling of F832/33 are also required to validate its therapeutic potential. Interestingly, the closely related compound F832/36 was recently evaluated *in vivo* in mice for its ability to protect against liver ischemia/reperfusion injury by inhibiting mitochondrial CypD, and it demonstrated efficacy (21).

In conclusion, our findings demonstrate the feasibility of tailoring SMCypIs to enhance their antiviral breadth and potency. This establishes them as promising, adaptable tools for combating current and future respiratory virus outbreaks. Our results support the further development of F832/33 as a promising broad-spectrum antiviral candidate with the potential to be used in the front-line treatment of both endemic and emerging respiratory pathogens.

## Data Availability

All data generated or analyzed in the current study will be made available from the corresponding authors on reasonable request.

## SUPPLEMENTAL MATERIAL

Supplemental material aac.01721-25-s0001.pdf Spectral Characterization of SMCypIs lead compounds.

ASM does not own the copyrights to Supplemental Material that may be linked to, or accessed through, an article. The authors have granted ASM a non-exclusive, world-wide license to publish the Supplemental Material files. Please contact the corresponding author directly for reuse.
