# Supplementary material for: Optimization of the antiviral spectrum of cyclophilin inhibitors targeting respiratory viruses
Source: Antimicrob Agents Chemother. 2026 Jul 17;70(8):e01721-25. doi: 10.1128/aac.01721-25 (PMC13435934; doi:10.1128/aac.01721-25)
Supplement: Supplementary file 1 [file aac.01721-25-s0001.pdf]

Certificate of Analysis

Chemical Name: /
Reference Name: F832
Batch Number: JFG23-2CF2
CAS Number: /
Origin of Country: (France)
Structure:

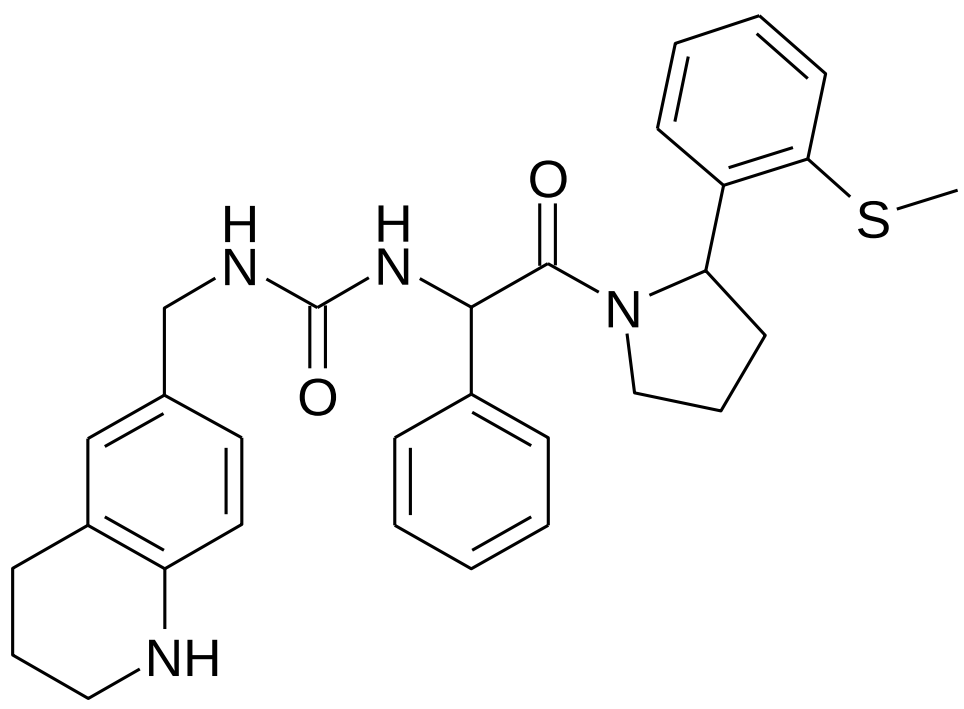

Item	Specification	Result
Purity (HPLC)	≥95.0%	>99%
Mass Spectrometry	514.70	515.8 (M+1)

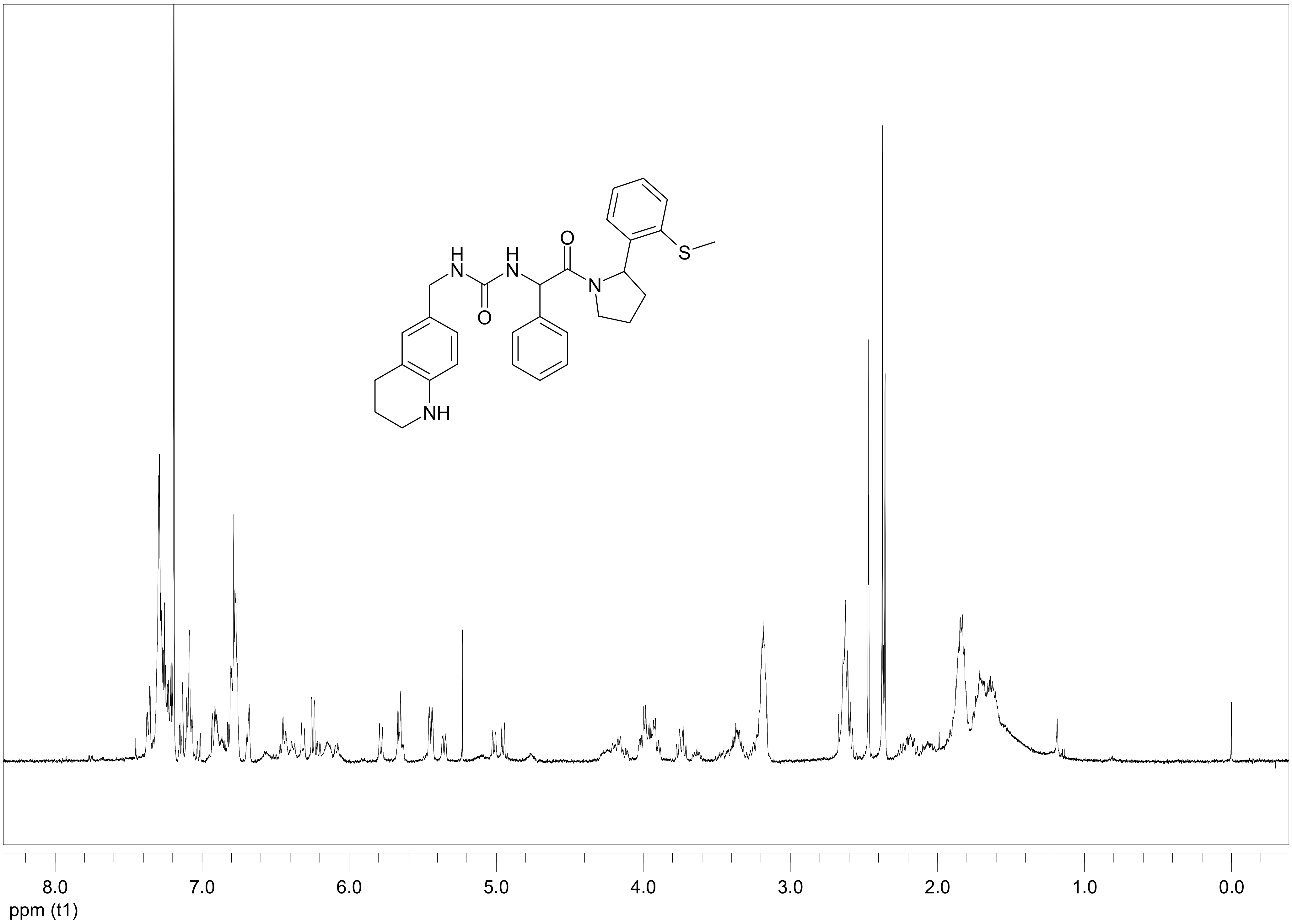

H
N
H
N
O
N
O
S
NH
8.0
7.0
6.0
5.0
4.0
3.0
2.0
1.0
0.0
ppm (t1)

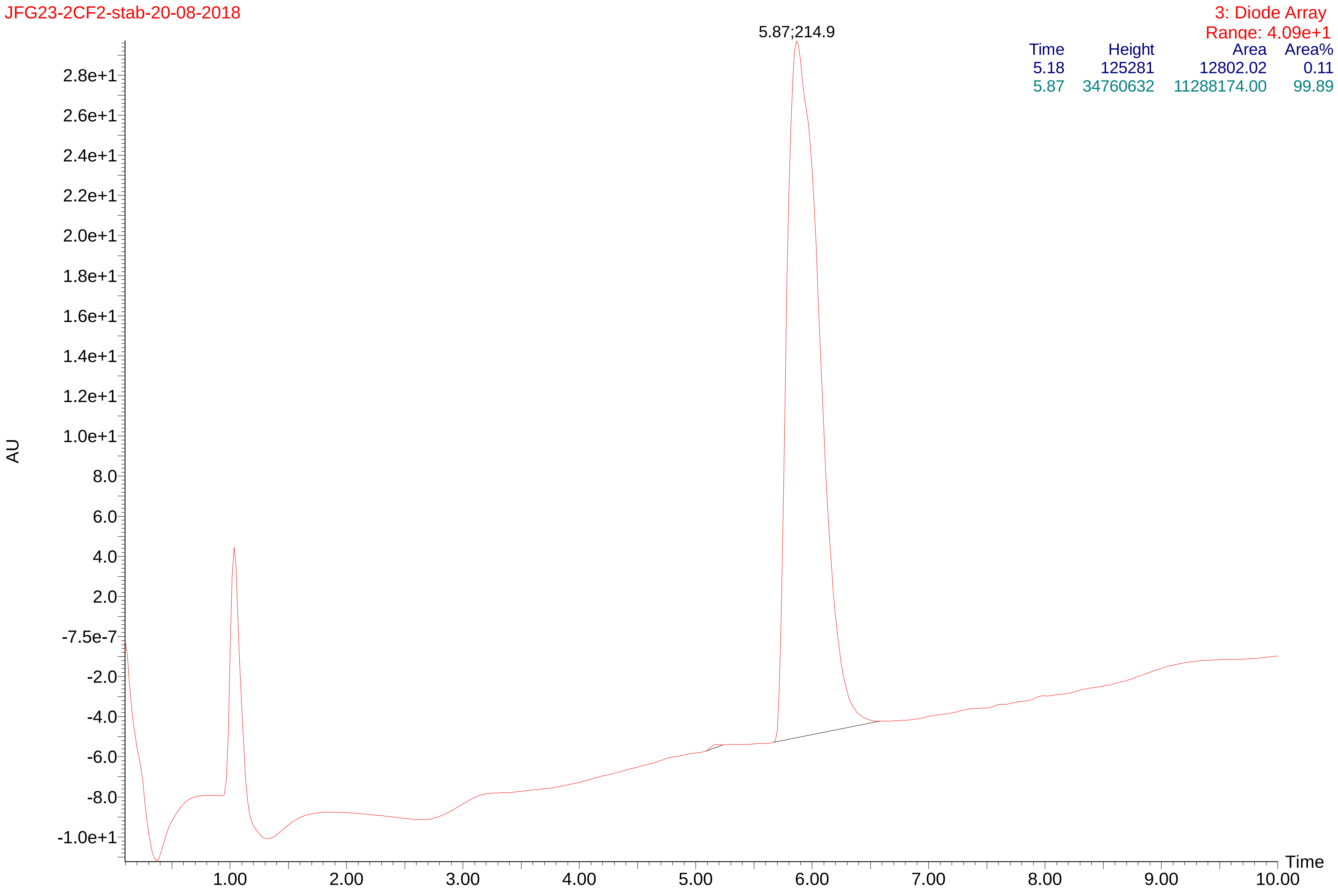

JFG23-2CF2-stab-20-08-2018
3: Diode Array
Range: 4.09e+1
5.87;214.9
Time Height Area Area%
5.18 125281 12802.02 0.11
5.87 34760632 11288174.00 99.89
AU
2.8e+1
2.6e+1
2.4e+1
2.2e+1
2.0e+1
1.8e+1
1.6e+1
1.4e+1
1.2e+1
1.0e+1
8.0
6.0
4.0
2.0
-7.5e-7
-2.0
-4.0
-6.0
-8.0
-1.0e+1
1.00
2.00
3.00
4.00
5.00
6.00
7.00
8.00
9.00
10.00
Time

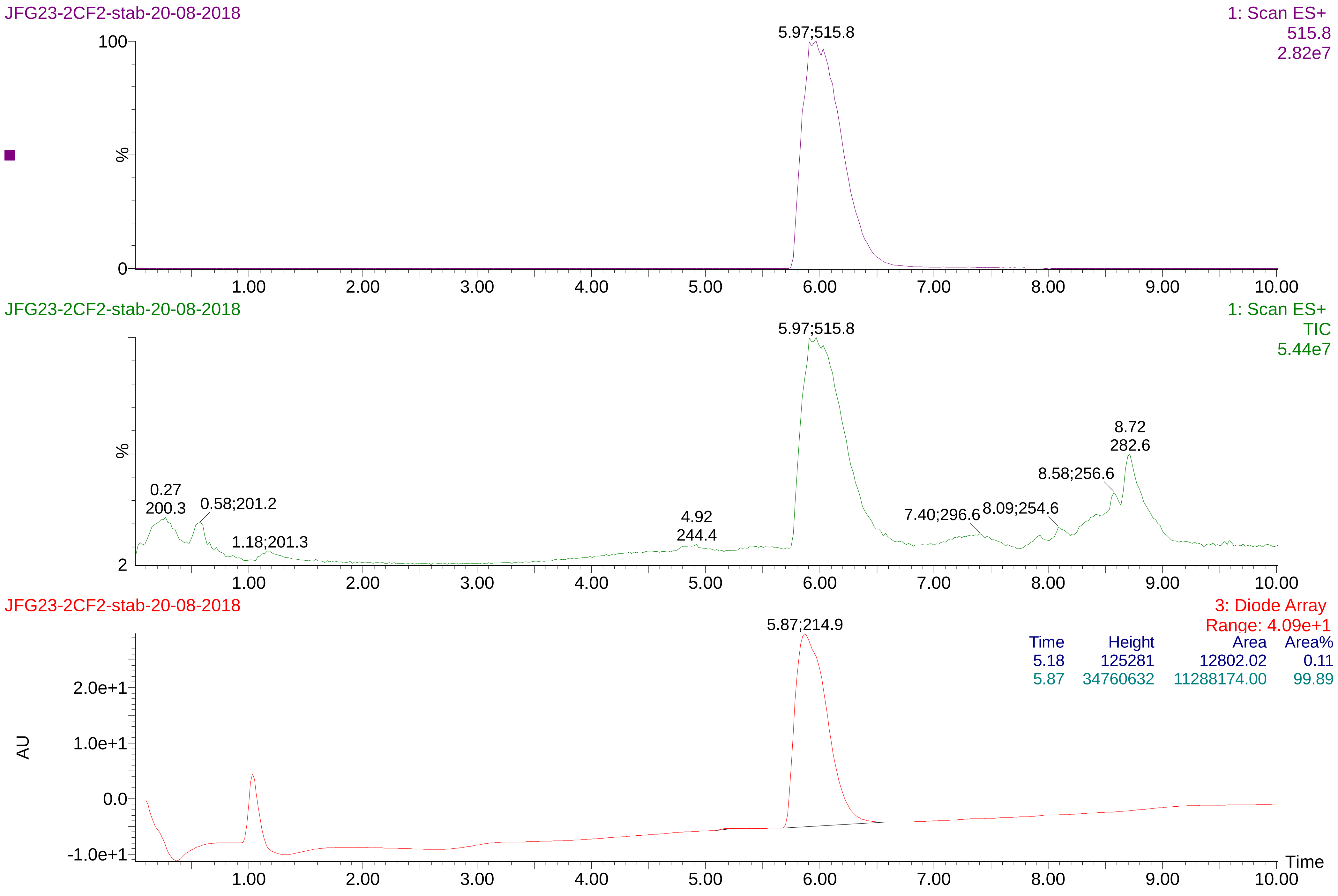
JFG23-2CF2-stab-20-08-2018
1: Scan ES+
515.8
2.82e7
5.97;515.8
100
%
0
1.00
2.00
3.00
4.00
5.00
6.00
7.00
8.00
9.00
10.00
JFG23-2CF2-stab-20-08-2018
1: Scan ES+
TIC
5.44e7
5.97;515.8
0.27
200.3
0.58;201.2
1.18;201.3
4.92
244.4
7.40;296.6
8.09;254.6
8.58;256.6
8.72
282.6
2
JFG23-2CF2-stab-20-08-2018
3: Diode Array
Range: 4.09e+1
5.87;214.9
Time Height Area Area%
5.18 125281 12802.02 0.11
5.87 34760632 11288174.00 99.89
AU
2.0e+1
1.0e+1
0.0
-1.0e+1
Time

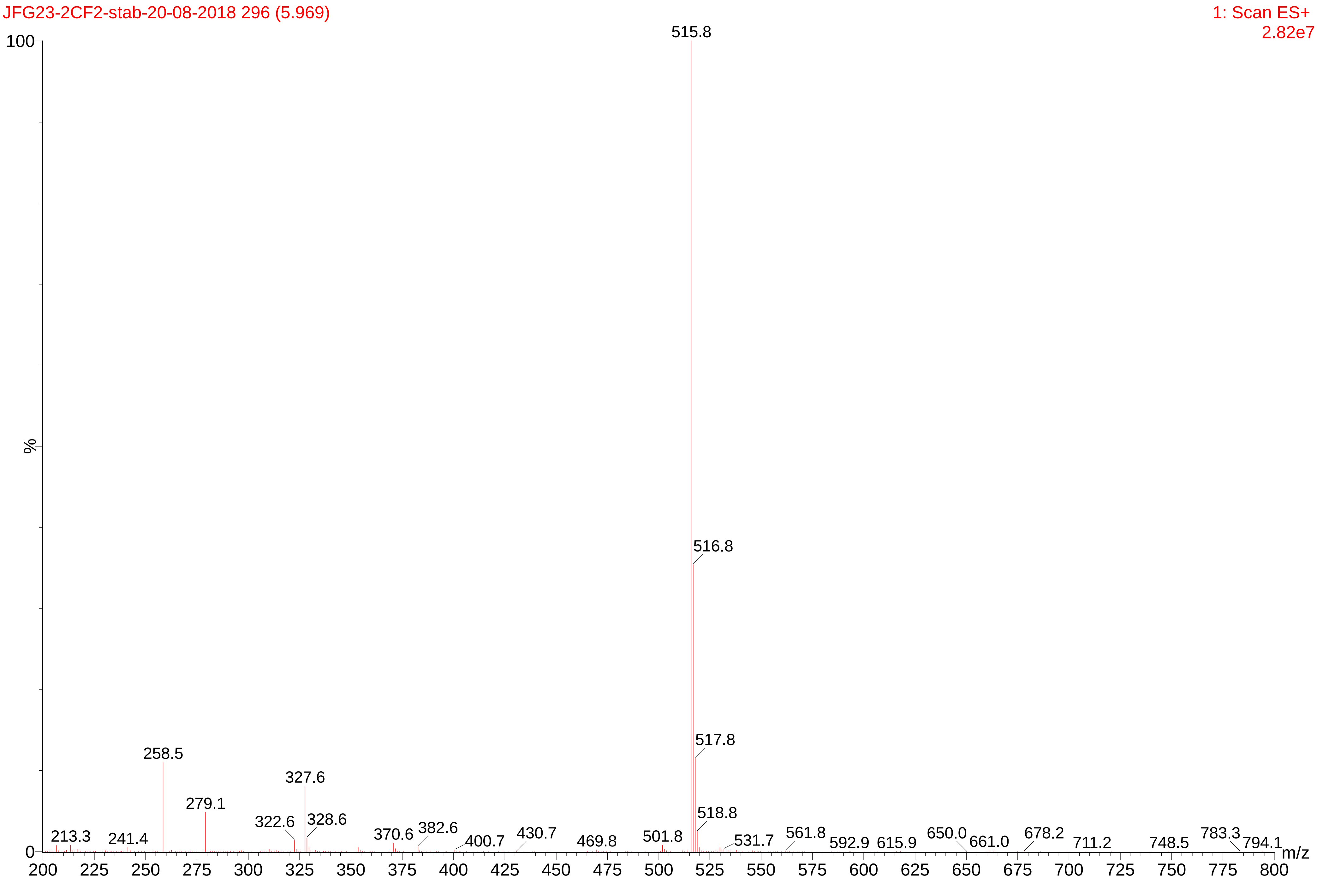
JFG23-2CF2-stab-20-08-2018 296 (5.969)
1: Scan ES+
2.82e7
515.8
516.8
517.8
518.8
258.5
327.6
279.1
322.6
328.6
213.3
241.4
370.6
382.6
400.7
430.7
469.8
501.8
531.7
561.8
592.9
615.9
650.0
661.0
678.2
711.2
748.5
783.3
794.1
%
100
0
m/z
200
225
250
275
300
325
350
375
400
425
450
475
500
525
550
575
600
625
650
675
700
725
750
775
800

Certificate of Analysis

Chemical Name:	/
Reference Name:	F833
Batch Number:	JFG30-1CF2
CAS Number:	/
Origin of Country:	AGV Discovery (France)
Structure:	

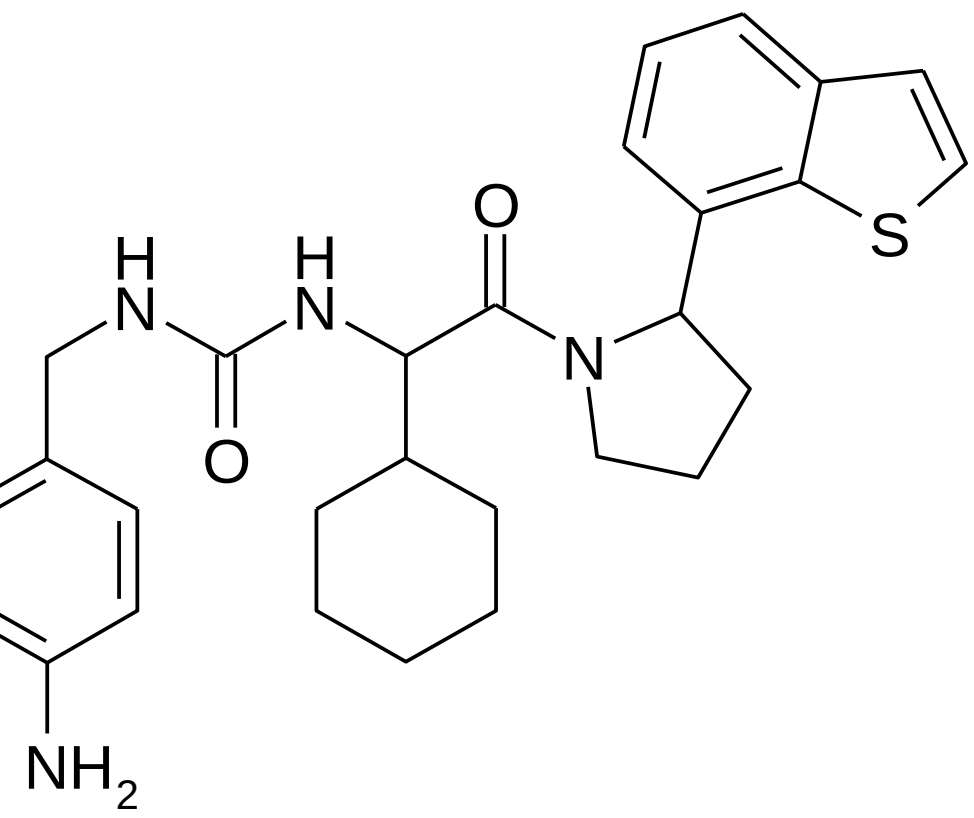

Item	Specification	Result
Purity (HPLC)	≥95.0%	>99%
Mass Spectrometry	490.67	491.8 (M+1)

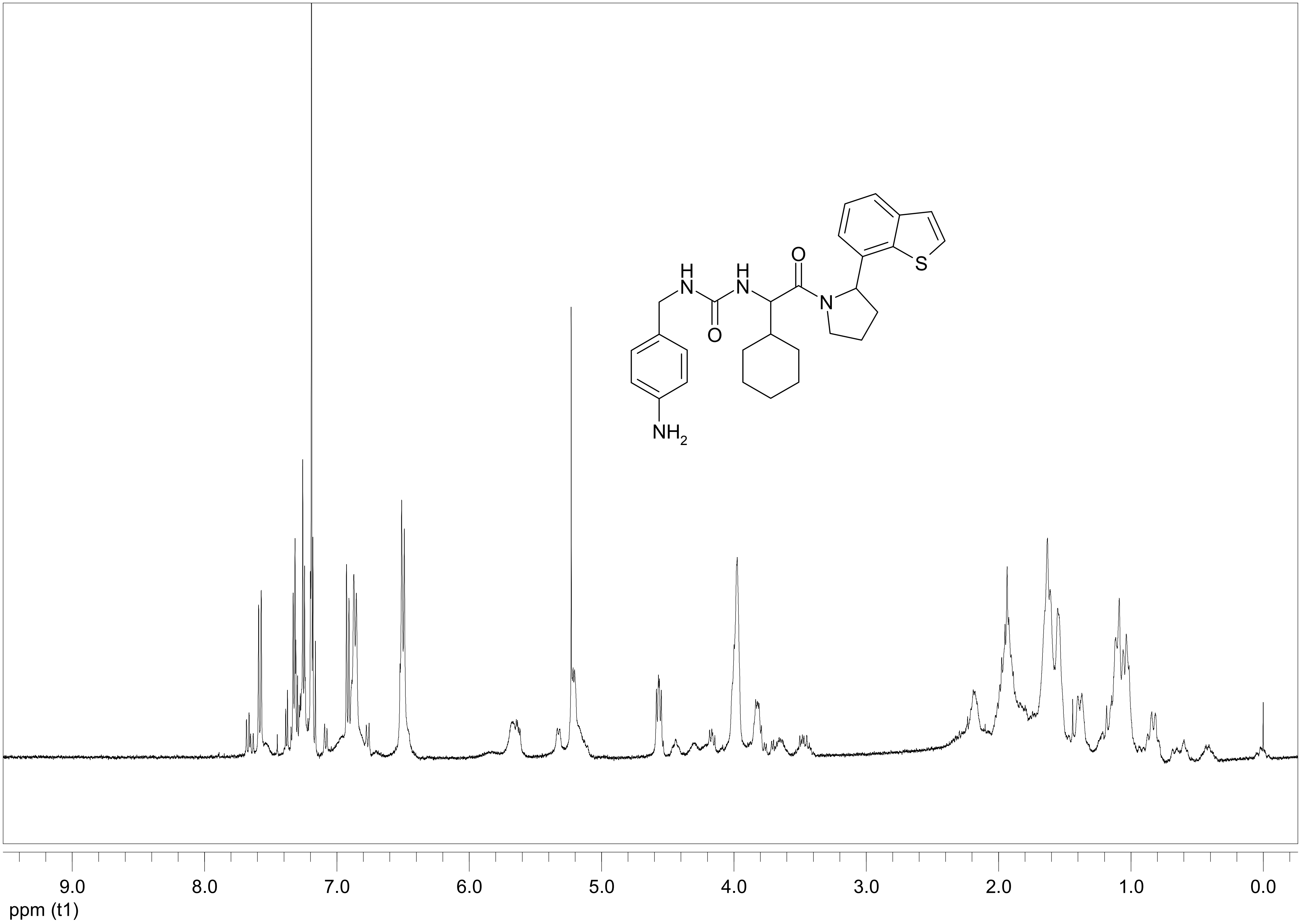

H
N
H
N
O
O
N
S
NH2
9.0
8.0
7.0
6.0
5.0
4.0
3.0
2.0
1.0
0.0
ppm (t1)

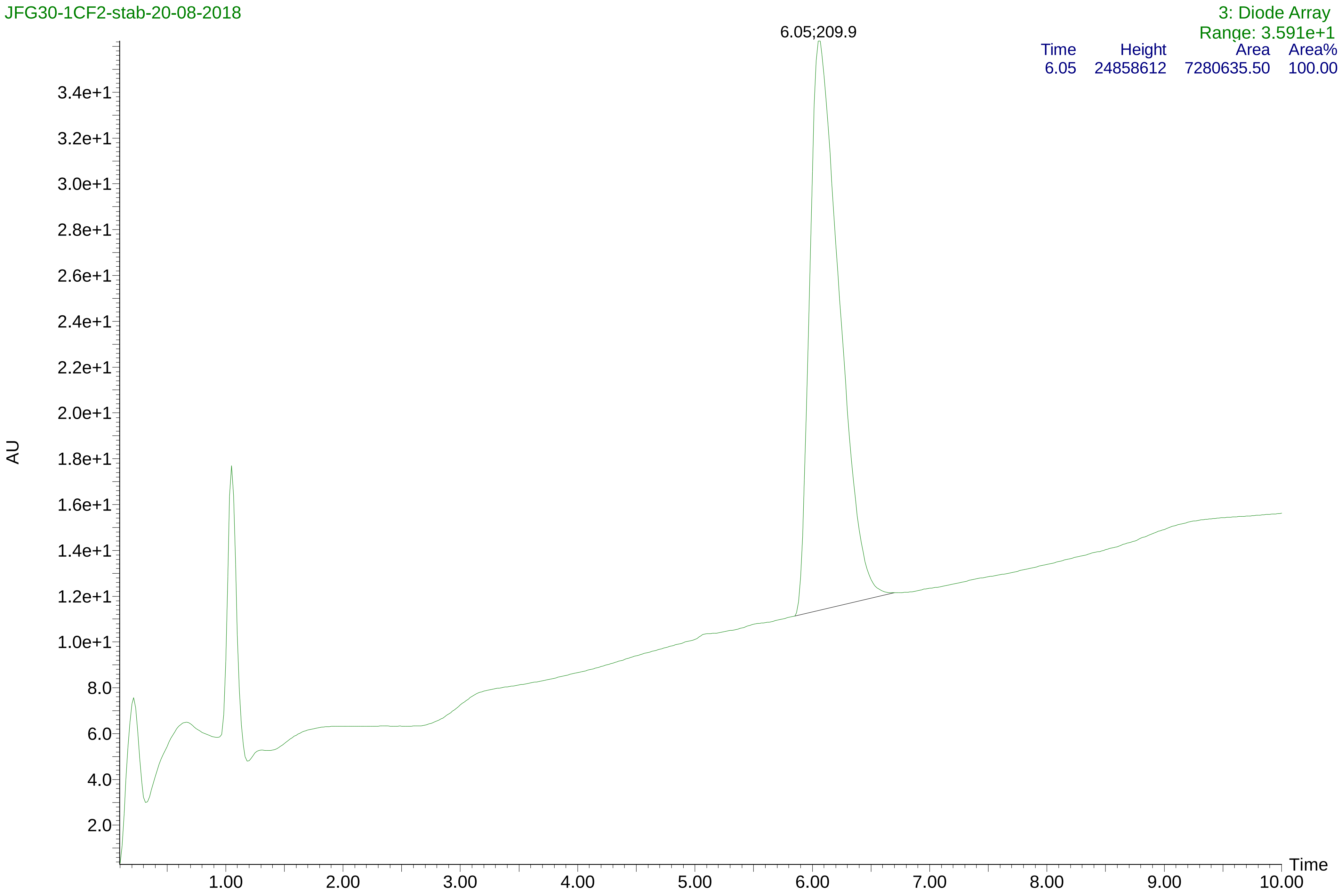
JFG30-1CF2-stab-20-08-2018
3: Diode Array
Range: 3.591e+1
6.05;209.9
Time Height Area Area%
6.05 24858612 7280635.50 100.00
AU
3.4e+1
3.2e+1
3.0e+1
2.8e+1
2.6e+1
2.4e+1
2.2e+1
2.0e+1
1.8e+1
1.6e+1
1.4e+1
1.2e+1
1.0e+1
8.0
6.0
4.0
2.0
1.00
2.00
3.00
4.00
5.00
6.00
7.00
8.00
9.00
10.00
Time

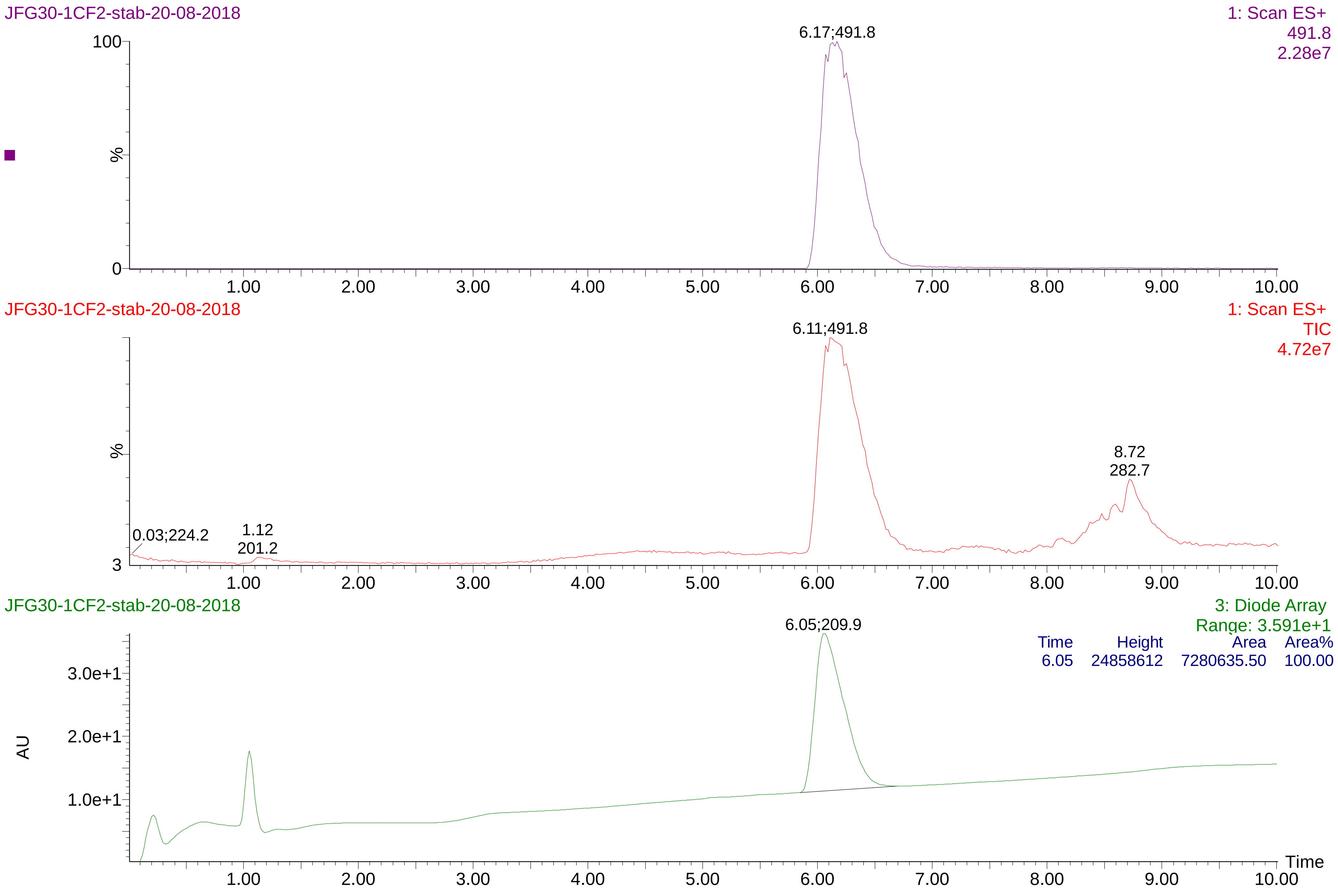

JFG30-1CF2-stab-20-08-2018
1: Scan ES+
491.8
2.28e7
6.17;491.8
100
%
0
1.00
2.00
3.00
4.00
5.00
6.00
7.00
8.00
9.00
10.00
JFG30-1CF2-stab-20-08-2018
1: Scan ES+
TIC
4.72e7
6.11;491.8
8.72
282.7
0.03;224.2
1.12
201.2
%
3
JFG30-1CF2-stab-20-08-2018
3: Diode Array
Range: 3.591e+1
Time Height Area Area%
6.05 24858612 7280635.50 100.00
6.05;209.9
3.0e+1
2.0e+1
1.0e+1
AU
Time

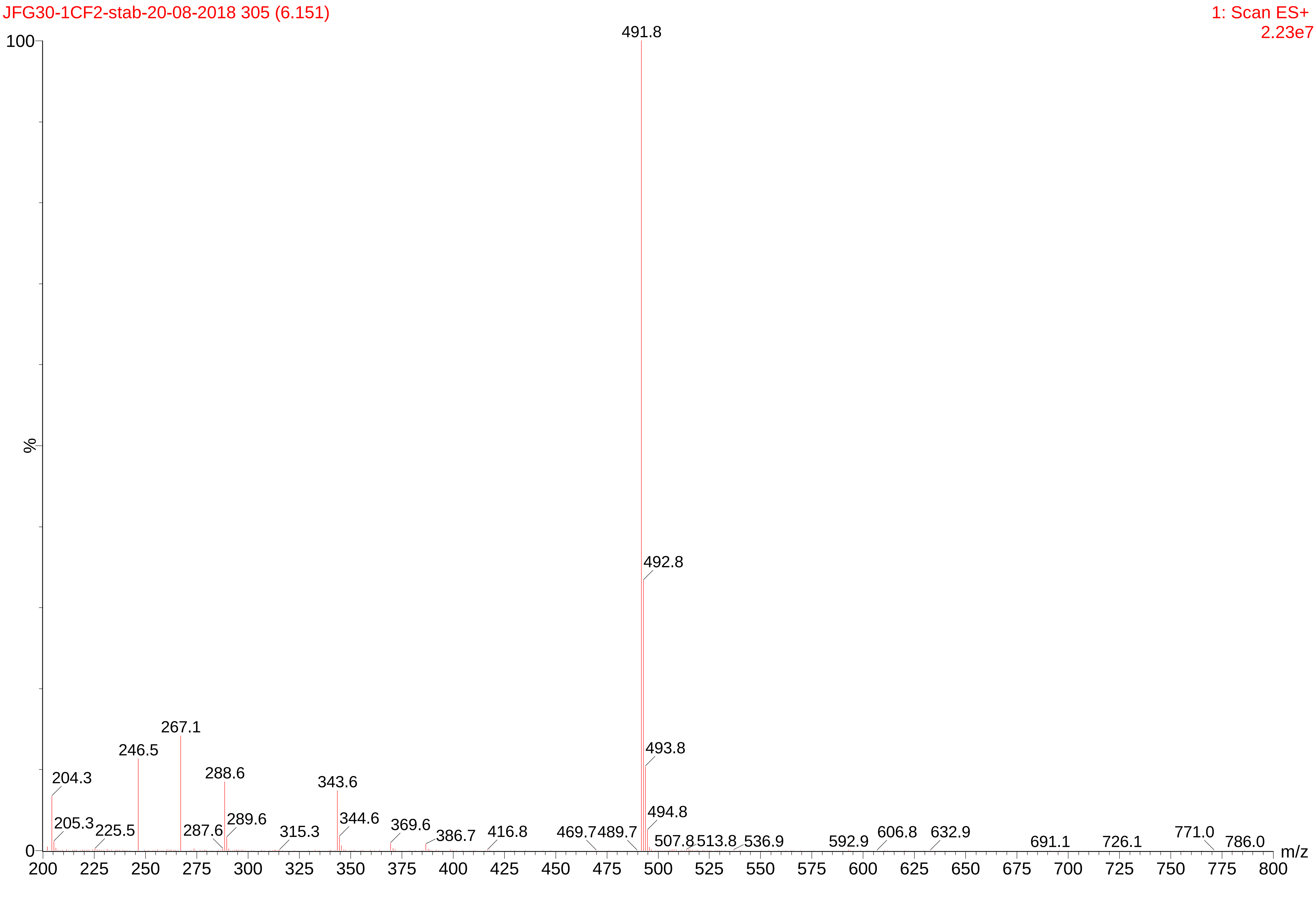

JFG30-1CF2-stab-20-08-2018 305 (6.151)
1: Scan ES+
2.23e7
491.8
492.8
493.8
494.8
267.1
246.5
288.6
204.3
343.6
205.3
225.5
287.6
289.6
315.3
344.6
369.6
386.7
416.8
469.7
489.7
507.8
513.8
536.9
592.9
606.8
632.9
691.1
726.1
771.0
786.0
100
%
0
200
225
250
275
300
325
350
375
400
425
450
475
500
525
550
575
600
625
650
675
700
725
750
775
800
m/z

Certificate of Analysis

Chemical Name: /
Reference Name: F836
Batch Number: JFG24-2DF1
CAS Number: /
Origin of Country: (France)
Structure:

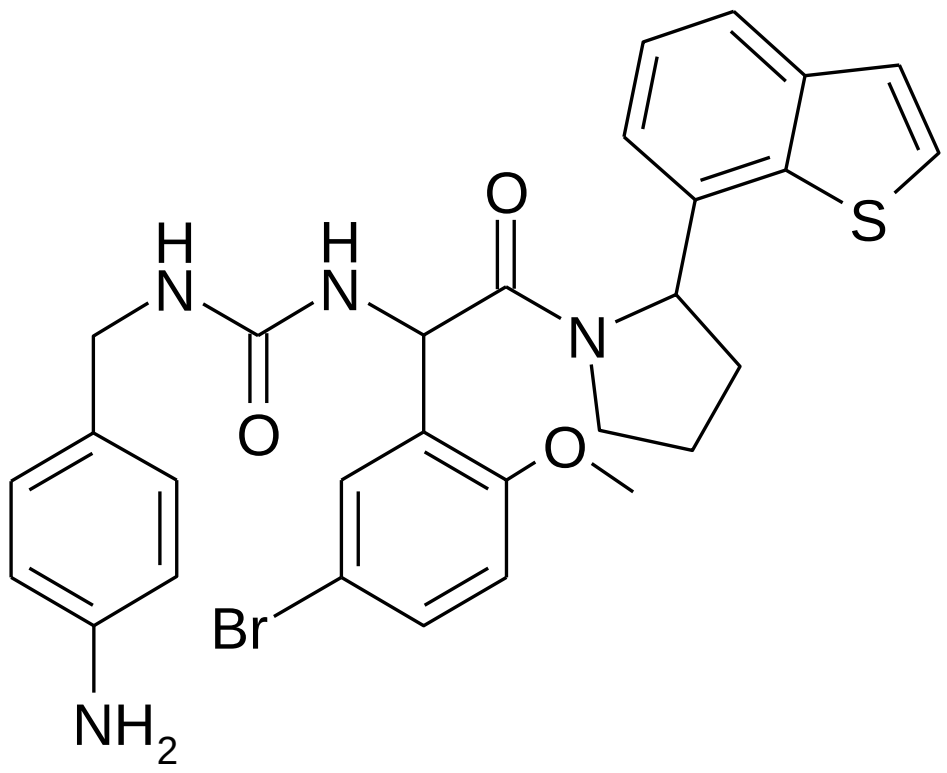

Item	Specification	Result
Purity (HPLC)	≥95.0%	>99%
Mass Spectrometry	593.55	593.7 ; 595.7 (M ; M+2)

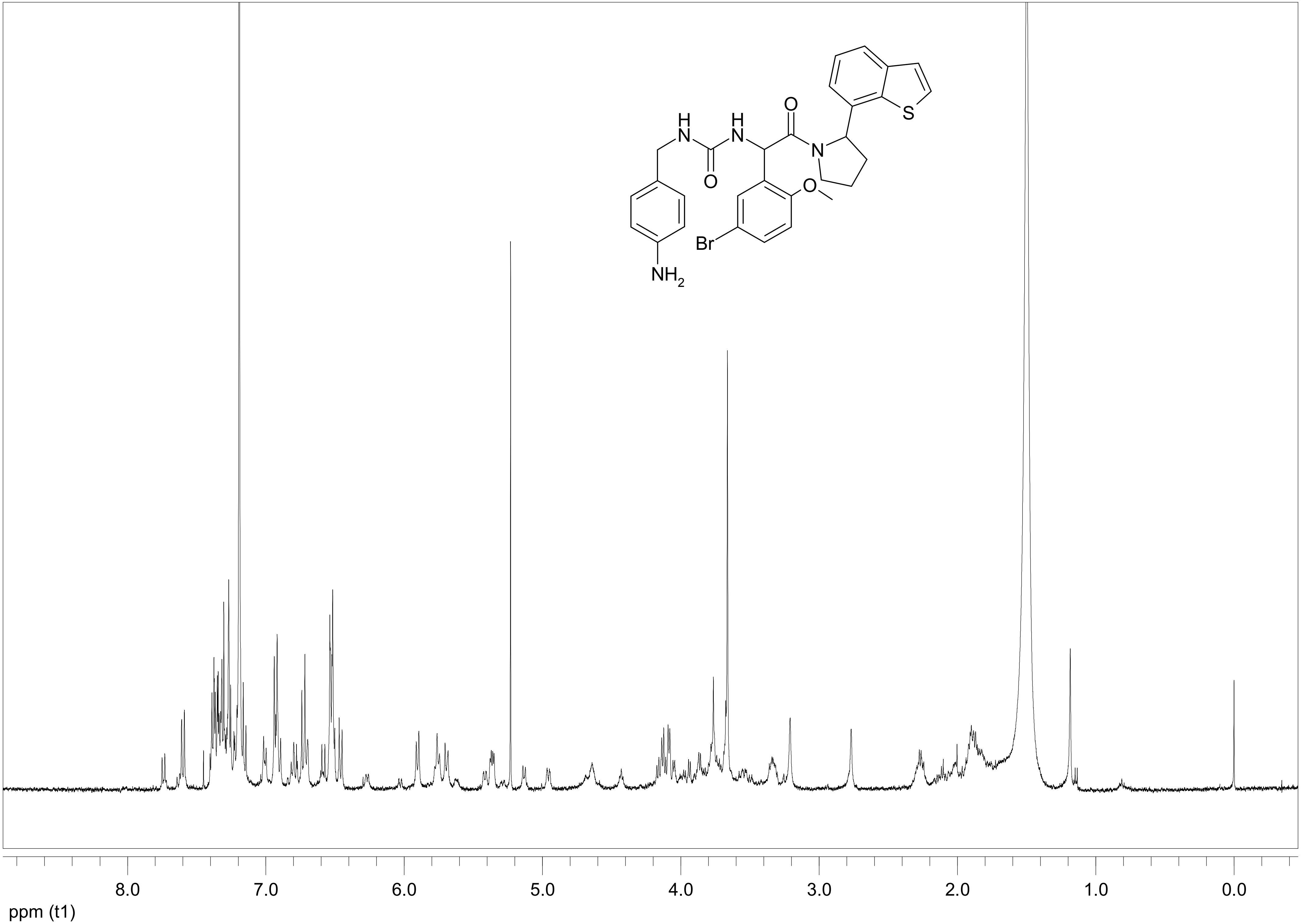

H
N
H
N
O
O
N
S
O
Br
NH2
8.0
7.0
6.0
5.0
4.0
3.0
2.0
1.0
0.0
ppm (t1)

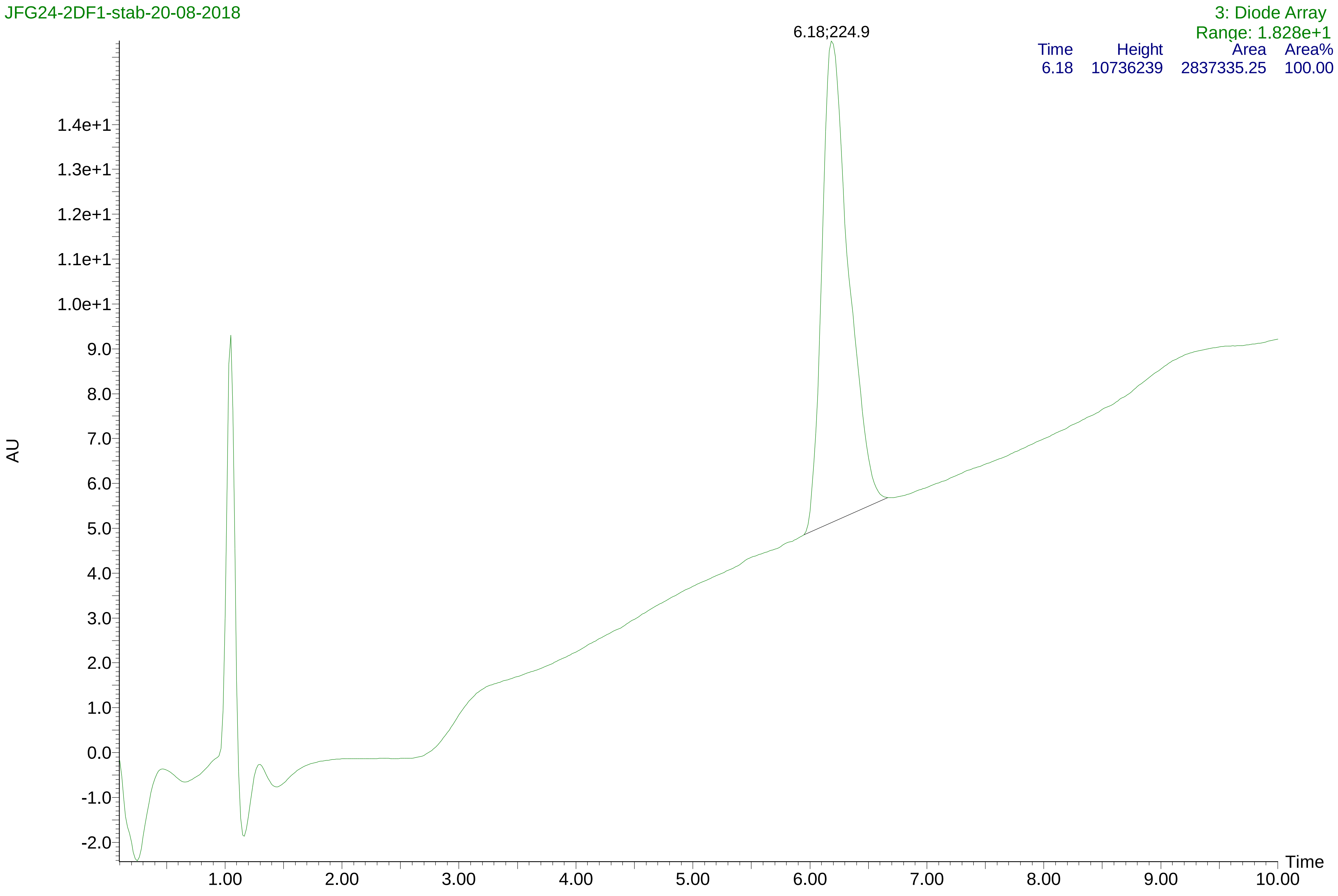

JFG24-2DF1-stab-20-08-2018
3: Diode Array
Range: 1.828e+1
6.18;224.9
Time Height Area Area%
6.18 10736239 2837335.25 100.00
AU
1.4e+1
1.3e+1
1.2e+1
1.1e+1
1.0e+1
9.0
8.0
7.0
6.0
5.0
4.0
3.0
2.0
1.0
0.0
-1.0
-2.0
1.00
2.00
3.00
4.00
5.00
6.00
7.00
8.00
9.00
10.00
Time

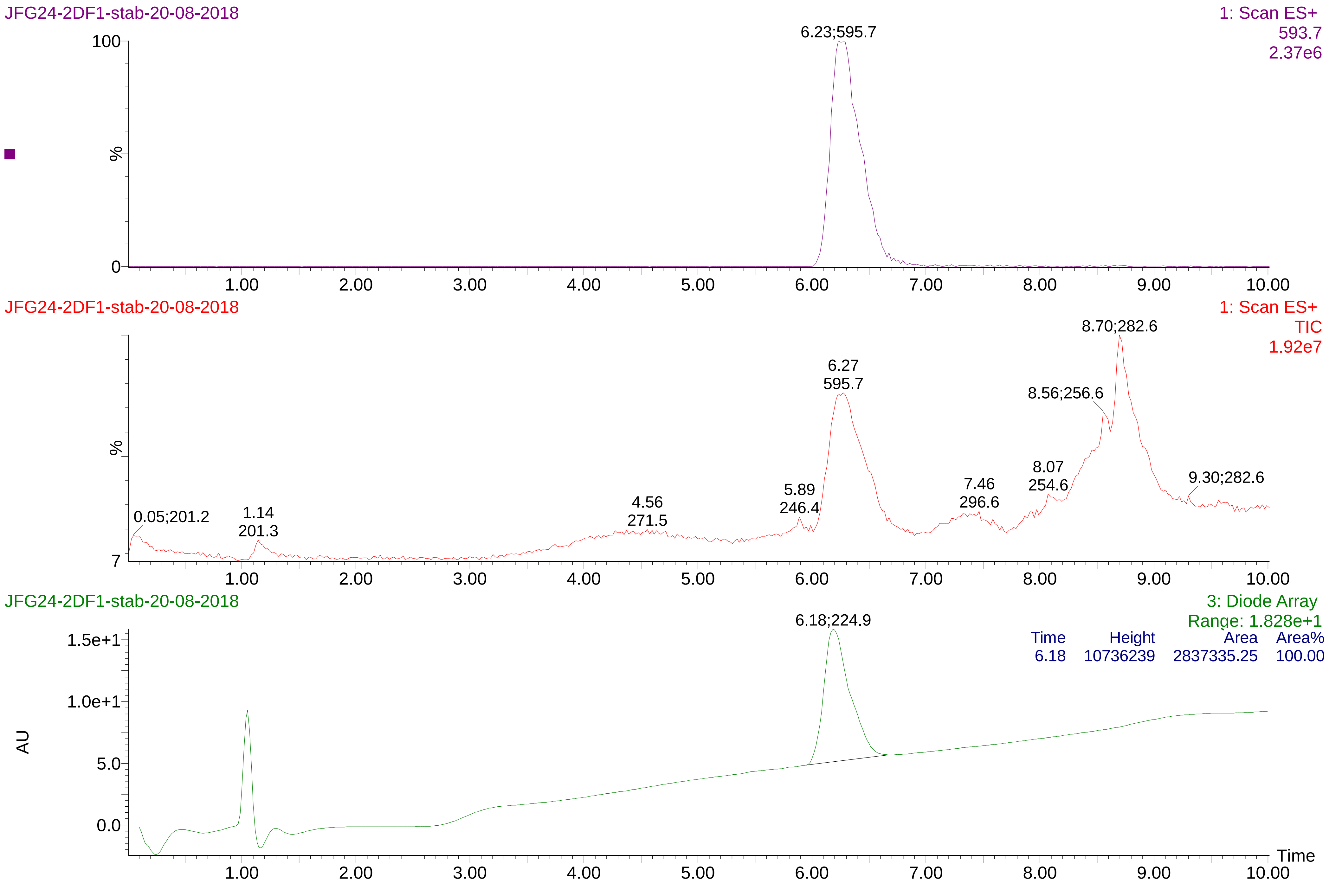

JFG24-2DF1-stab-20-08-2018
1: Scan ES+
593.7
2.37e6
6.23;595.7
100
%
0
1.00
2.00
3.00
4.00
5.00
6.00
7.00
8.00
9.00
10.00
JFG24-2DF1-stab-20-08-2018
1: Scan ES+
TIC
1.92e7
8.70;282.6
6.27
595.7
8.56;256.6
8.07
254.6
7.46
296.6
9.30;282.6
5.89
246.4
4.56
271.5
0.05;201.2
1.14
201.3
%
7
JFG24-2DF1-stab-20-08-2018
3: Diode Array
Range: 1.828e+1
6.18;224.9
Time Height Area Area%
6.18 10736239 2837335.25 100.00
1.5e+1
1.0e+1
5.0
0.0
AU
Time

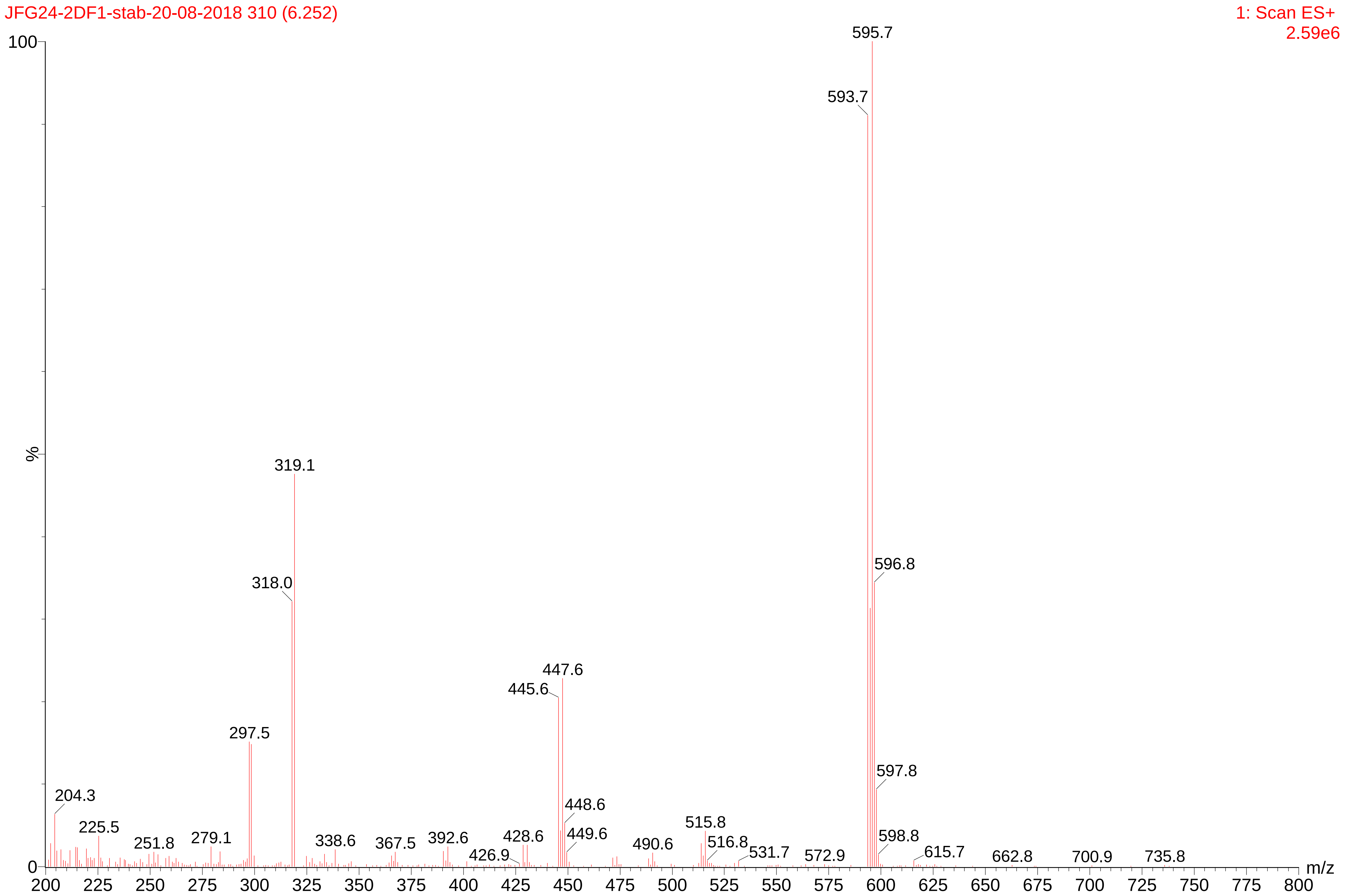

JFG24-2DF1-stab-20-08-2018 310 (6.252)
1: Scan ES+
2.59e6
100
%
0
595.7
593.7
319.1
318.0
596.8
447.6
445.6
297.5
597.8
204.3
448.6
225.5
251.8
279.1
338.6
367.5
392.6
428.6
426.9
449.6
490.6
515.8
516.8
531.7
572.9
598.8
615.7
662.8
700.9
735.8
200
225
250
275
300
325
350
375
400
425
450
475
500
525
550
575
600
625
650
675
700
725
750
775
800
m/z

Certificate of Analysis

Chemical Name: /
Reference Name: F83236
Batch Number: JFG26-1CF2
CAS Number: /
Origin of Country: (France)
Structure:

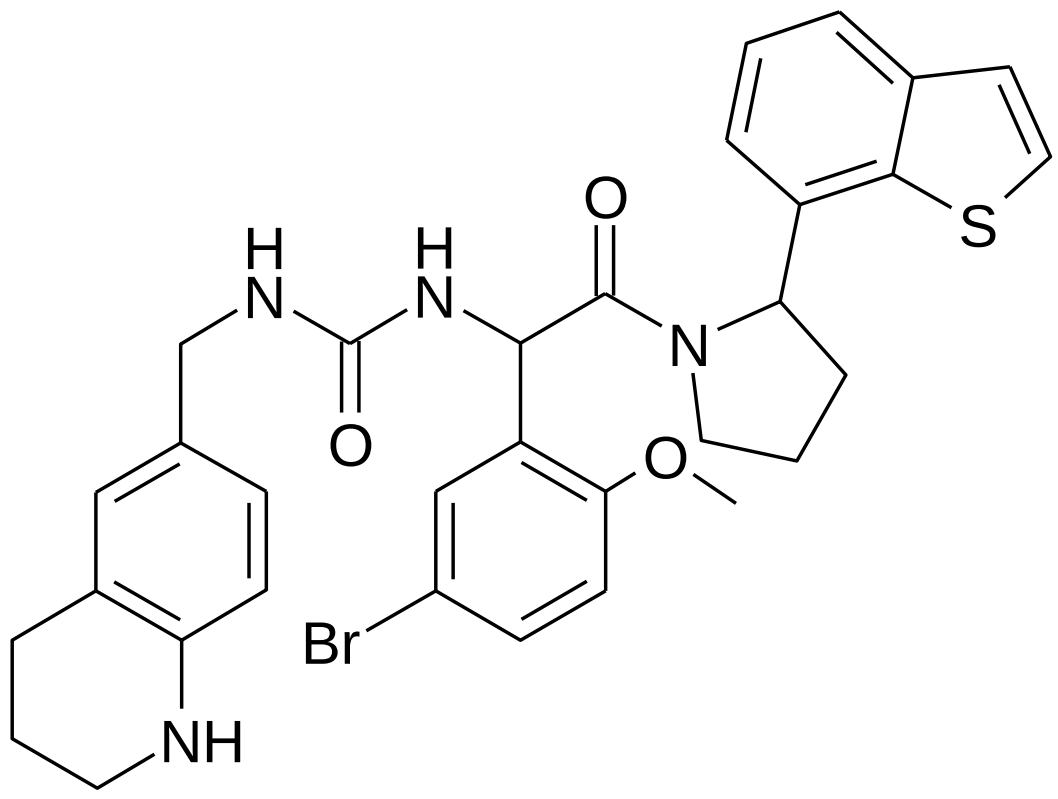

Item	Specification	Result
Purity (HPLC)	≥95.0%	>99%
Mass Spectrometry	633.61	633.8 ; 635.8 (M ; M+2)

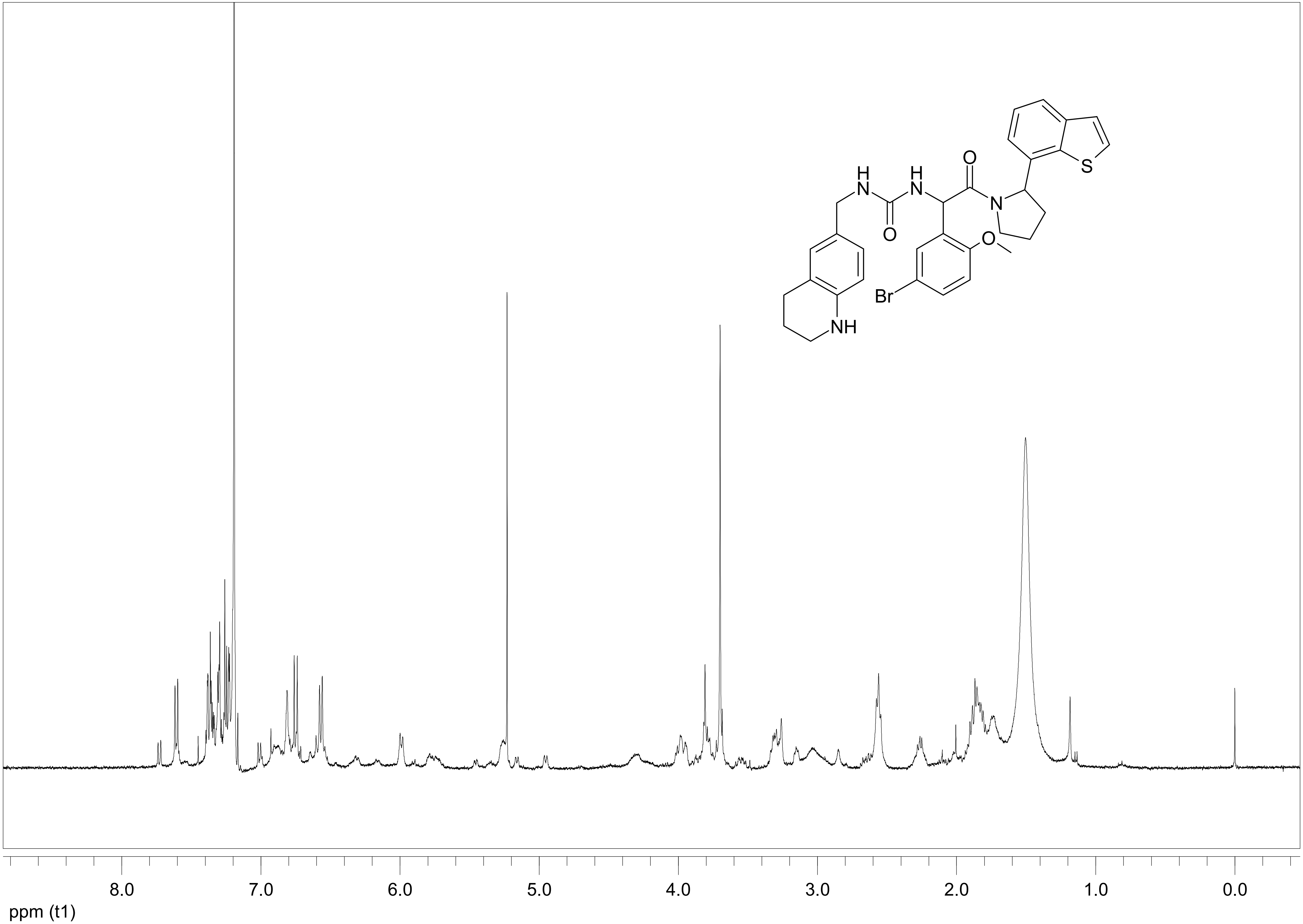

O
H
N
H
N
N
S
O
O
Br
NH
8.0
7.0
6.0
5.0
4.0
3.0
2.0
1.0
0.0
ppm (t1)

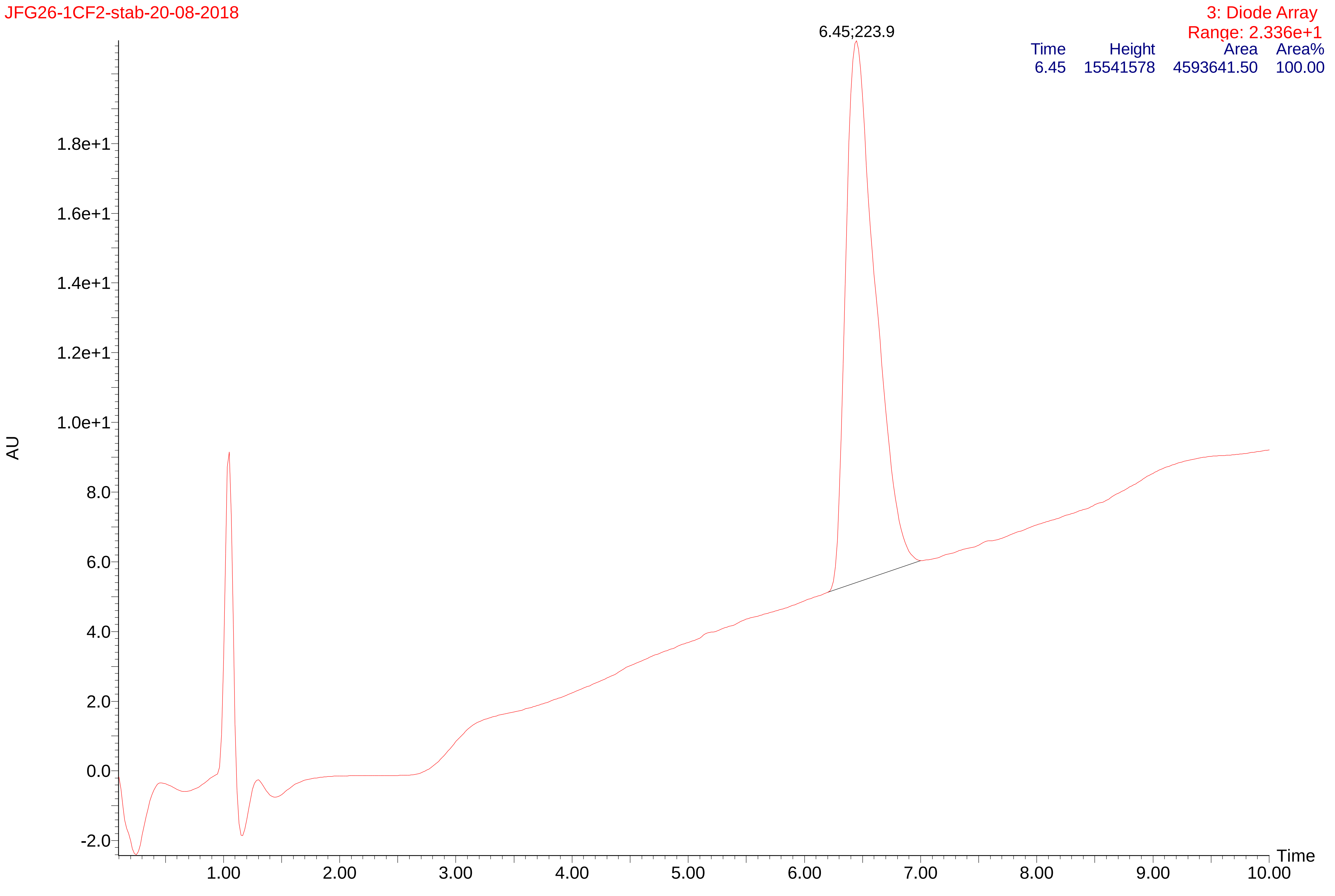
JFG26-1CF2-stab-20-08-2018
3: Diode Array
Range: 2.336e+1
6.45;223.9
Time Height Area Area%
6.45 15541578 4593641.50 100.00
AU
1.8e+1
1.6e+1
1.4e+1
1.2e+1
1.0e+1
8.0
6.0
4.0
2.0
0.0
-2.0
1.00
2.00
3.00
4.00
5.00
6.00
7.00
8.00
9.00
10.00
Time

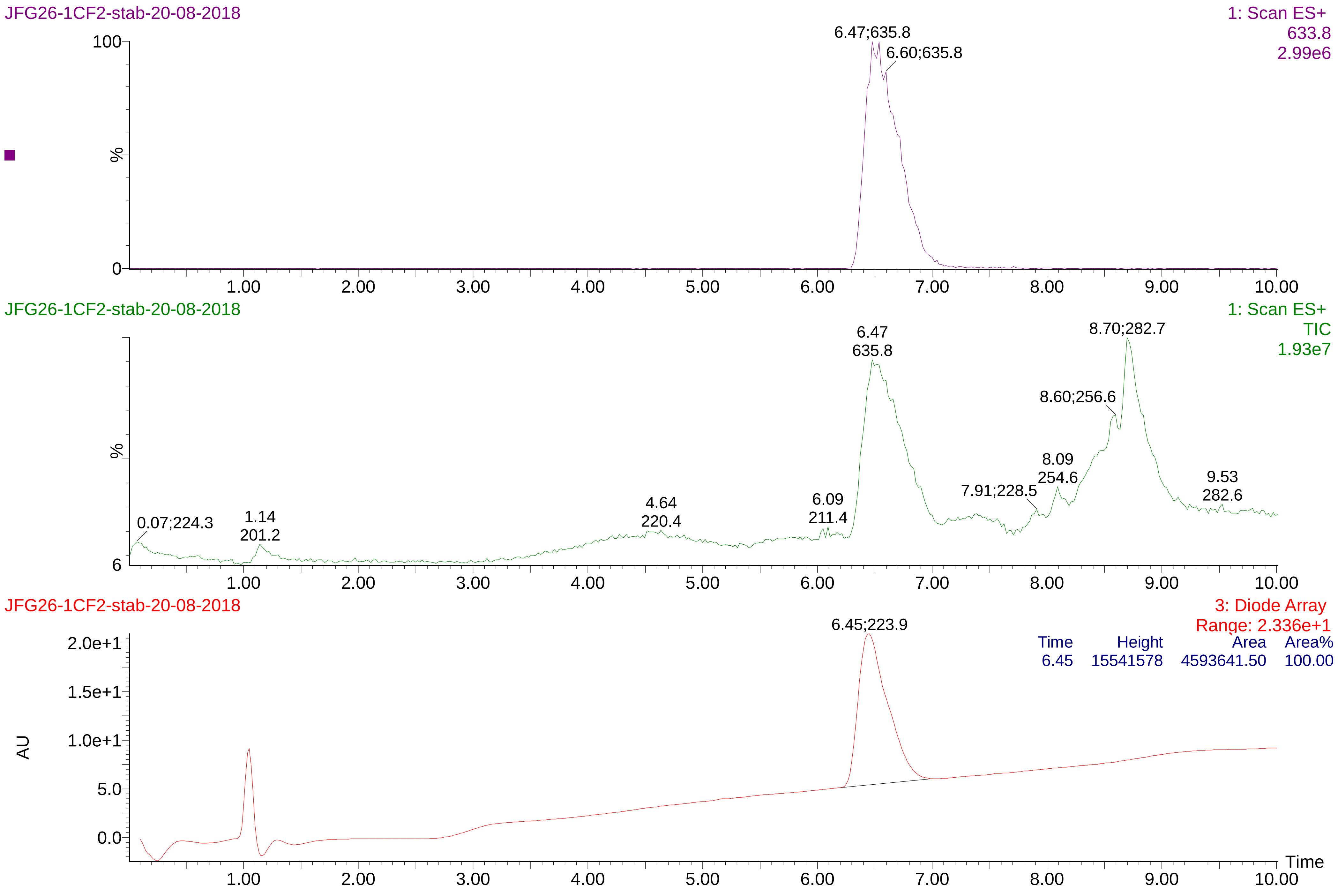

JFG26-1CF2-stab-20-08-2018
1: Scan ES+
633.8
2.99e6
6.47;635.8
6.60;635.8
100
%
0
1.00
2.00
3.00
4.00
5.00
6.00
7.00
8.00
9.00
10.00
JFG26-1CF2-stab-20-08-2018
1: Scan ES+
TIC
1.93e7
6.47
635.8
8.70;282.7
8.60;256.6
8.09
254.6
7.91;228.5
9.53
282.6
6.09
211.4
4.64
220.4
1.14
201.2
0.07;224.3
%
6
JFG26-1CF2-stab-20-08-2018
3: Diode Array
Range: 2.336e+1
6.45;223.9
Time Height Area Area%
6.45 15541578 4593641.50 100.00
AU
2.0e+1
1.5e+1
1.0e+1
5.0
0.0
Time

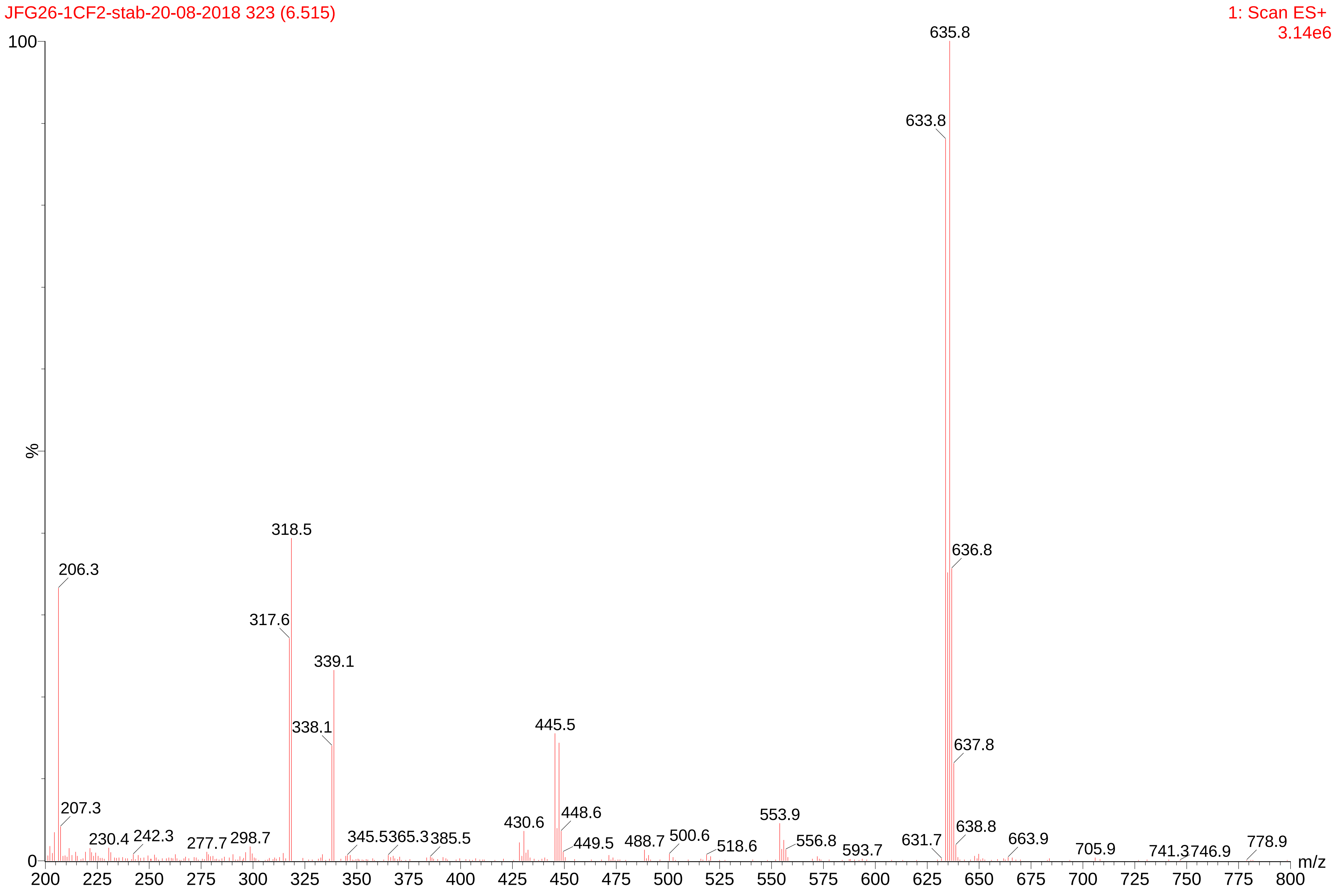

JFG26-1CF2-stab-20-08-2018 323 (6.515)
1: Scan ES+
3.14e6
100
%
0
635.8
633.8
636.8
637.8
638.8
631.7
663.9
705.9
741.3
746.9
778.9
318.5
317.6
339.1
338.1
206.3
207.3
230.4
242.3
277.7
298.7
345.5
365.3
385.5
430.6
445.5
448.6
449.5
488.7
500.6
518.6
553.9
556.8
593.7
200 225 250 275 300 325 350 375 400 425 450 475 500 525 550 575 600 625 650 675 700 725 750 775 800
m/z

Certificate of Analysis

Chemical Name: /
Reference Name: F83233
Batch Number: JFG32-1CF3
CAS Number: /
Origin of Country: (France)
Structure:

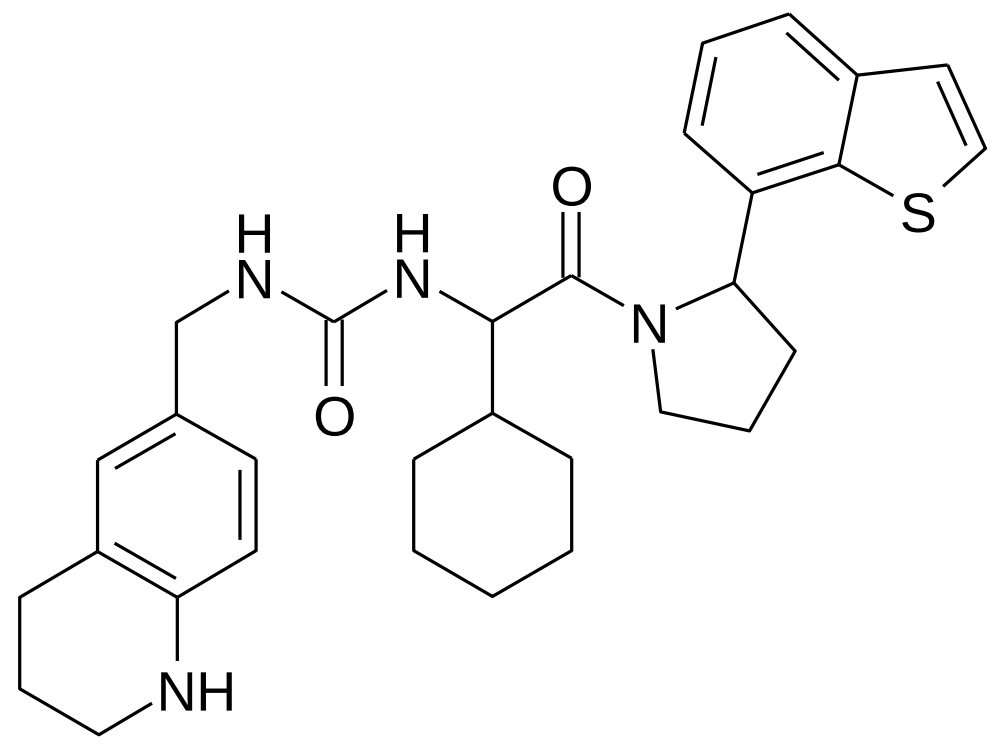

Item	Specification	Result
Purity (HPLC)	≥95.0%	>99%
Mass Spectrometry	530.74	531.9 (M+1)

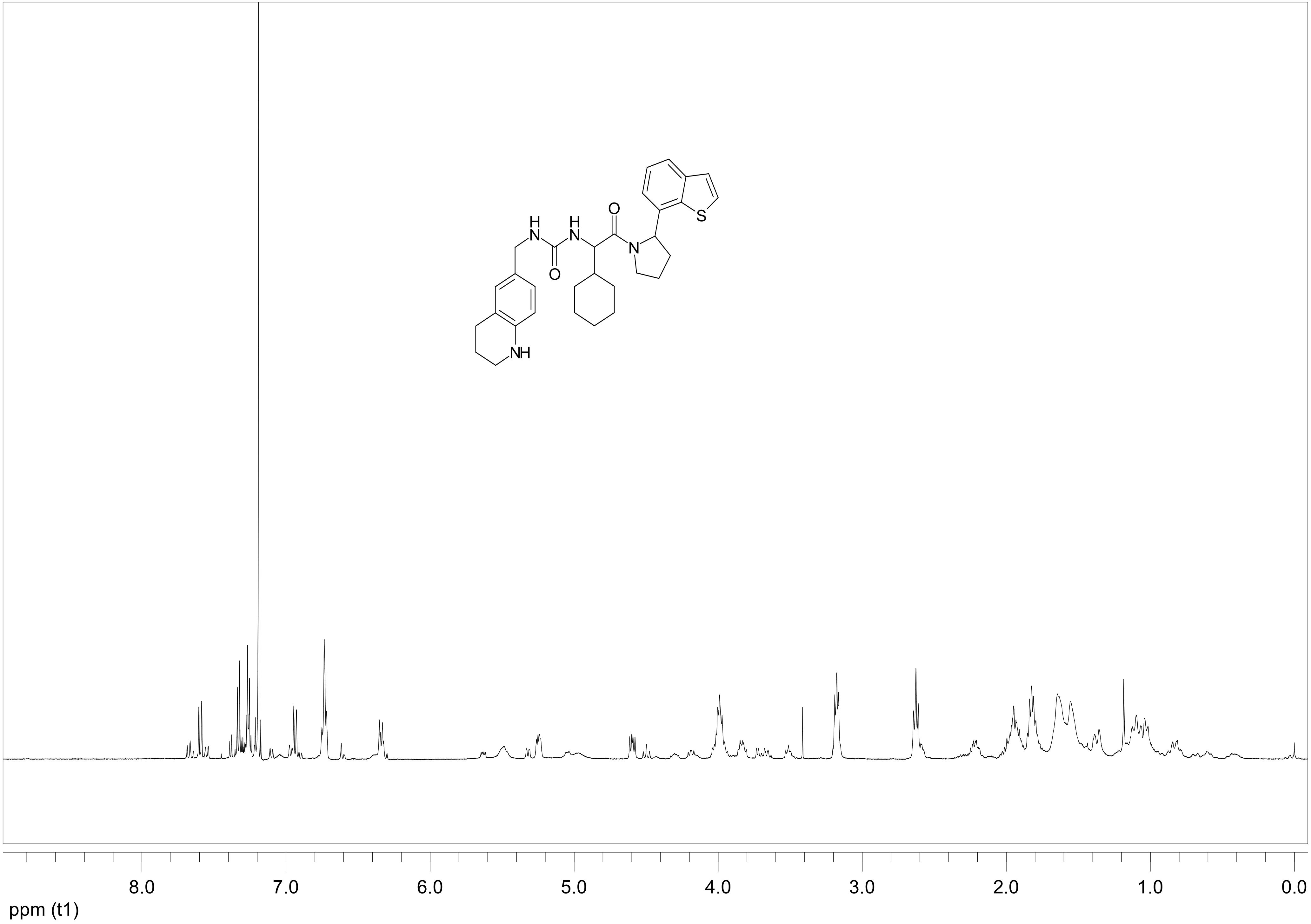
8.0
7.0
6.0
5.0
4.0
3.0
2.0
1.0
0.0
ppm (t1)

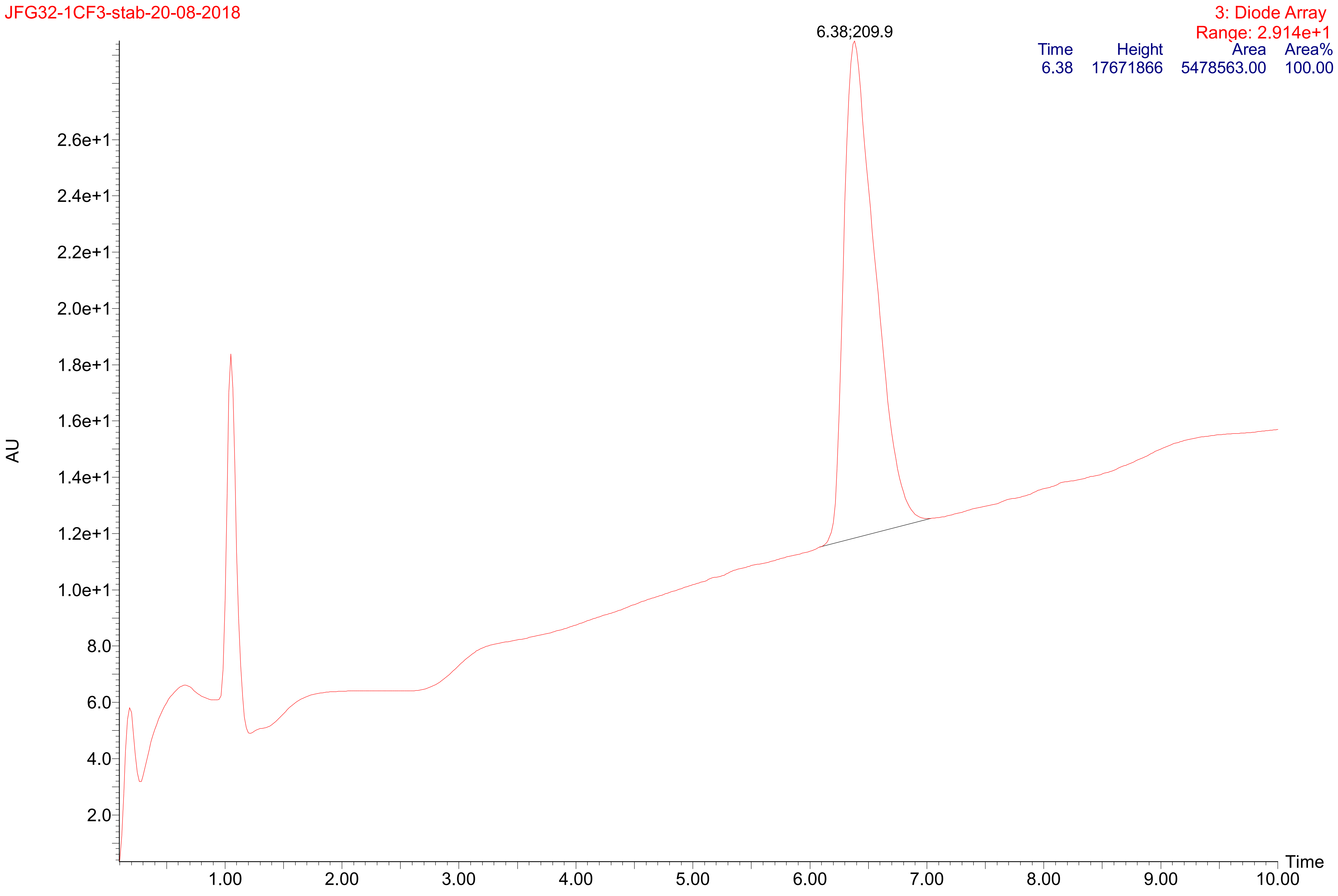

JFG32-1CF3-stab-20-08-2018
3: Diode Array
Range: 2.914e+1
6.38;209.9
Time Height Area Area%
6.38 17671866 5478563.00 100.00
AU
2.6e+1
2.4e+1
2.2e+1
2.0e+1
1.8e+1
1.6e+1
1.4e+1
1.2e+1
1.0e+1
8.0
6.0
4.0
2.0
1.00
2.00
3.00
4.00
5.00
6.00
7.00
8.00
9.00
10.00
Time

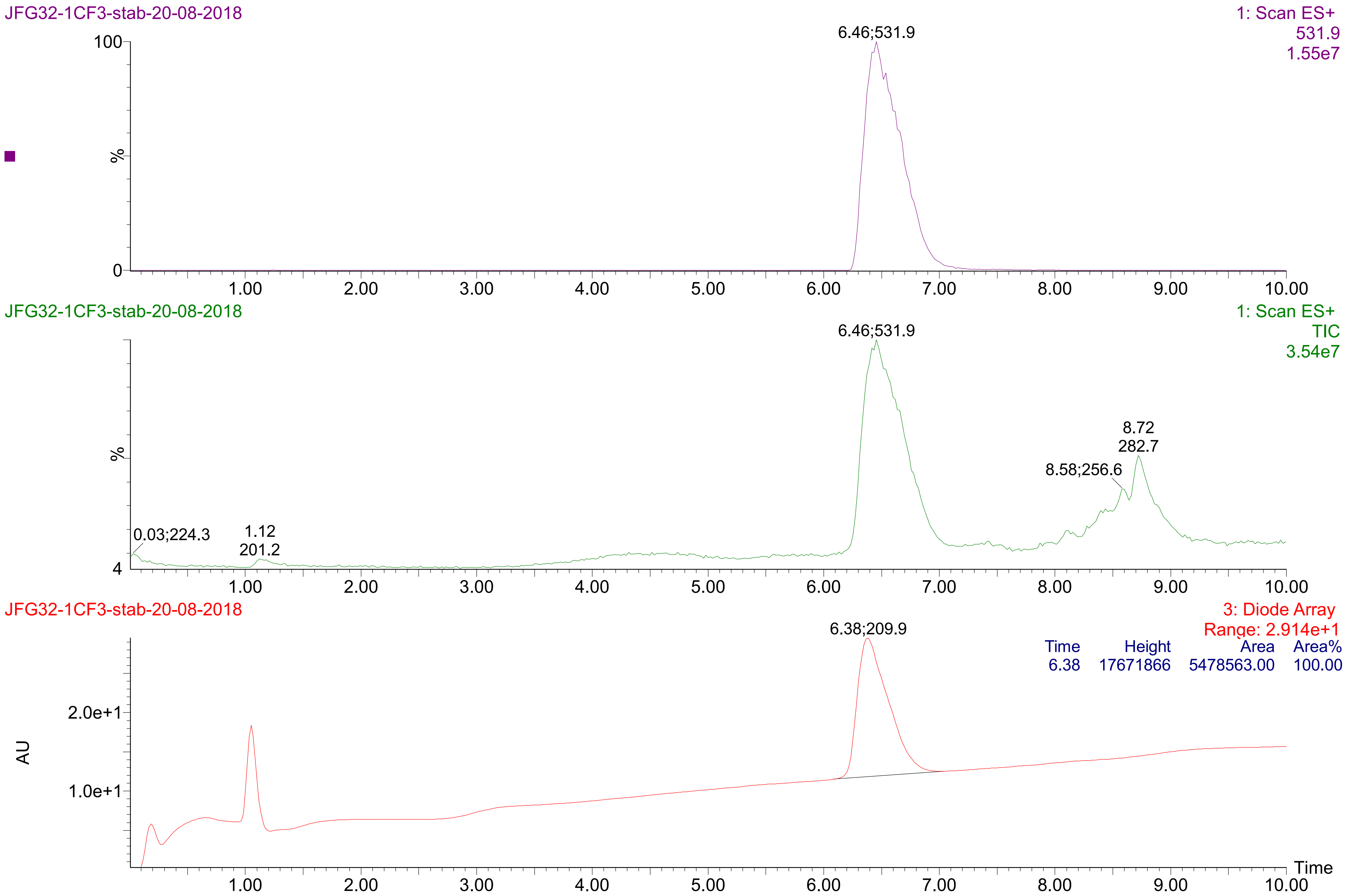
JFG32-1CF3-stab-20-08-2018
1: Scan ES+
531.9
1.55e7
6.46;531.9
100
%
0
1.00
2.00
3.00
4.00
5.00
6.00
7.00
8.00
9.00
10.00
JFG32-1CF3-stab-20-08-2018
1: Scan ES+
TIC
3.54e7
6.46;531.9
8.72
282.7
8.58;256.6
0.03;224.3
1.12
201.2
%
4
JFG32-1CF3-stab-20-08-2018
3: Diode Array
Range: 2.914e+1
6.38;209.9
Time
6.38
Height
17671866
Area
5478563.00
Area%
100.00
2.0e+1
1.0e+1
AU
Time

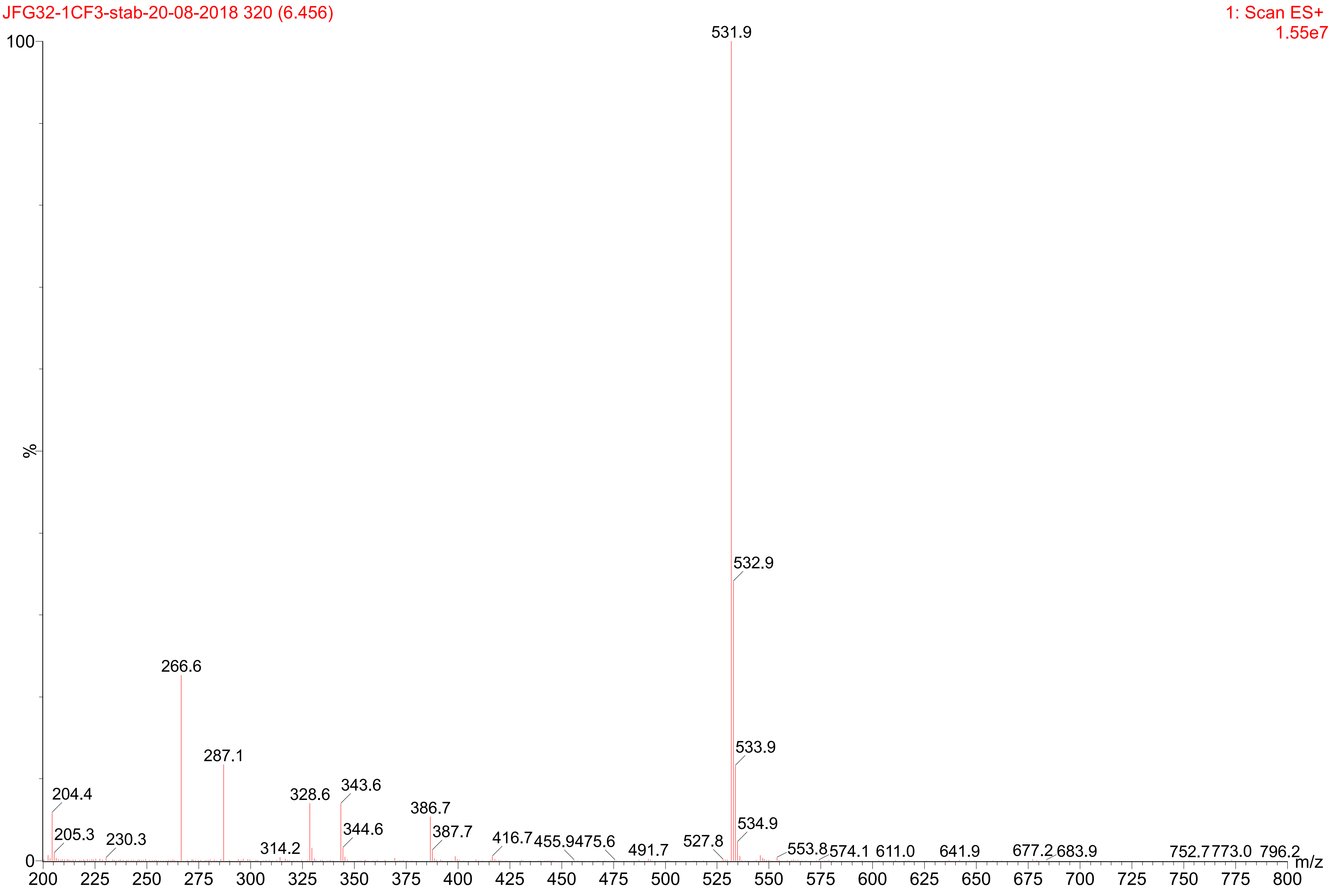

JFG32-1CF3-stab-20-08-2018 320 (6.456)
1: Scan ES+
1.55e7
100
%
0
531.9
532.9
533.9
534.9
266.6
287.1
204.4
205.3
230.3
314.2
328.6
343.6
344.6
386.7
387.7
416.7
455.9
475.6
491.7
527.8
553.8
574.1
611.0
641.9
677.2
683.9
752.7
773.0
796.2
m/z
200 225 250 275 300 325 350 375 400 425 450 475 500 525 550 575 600 625 650 675 700 725 750 775 800

Supplementary material

Spectral characterization of SMCypIs lead compounds

Chemical structures, purity (HPLC), NMR and mass spectrometry analysis of F832, F833, F836, F832/36 and F832/33.

Equipment and analytical methods used for the syntheses of examples

Flash chromatography:

Apparatus: Biotage SP with auto-collector and UV detection (2 wavelengths).

Normal phase columns: 120 g or 300 g Biotage external dry load cartridge kit, packed with Sigma-Aldrich 40-63 µm silica gel.

Liquid Chromatography:

Apparatus: Waters alliance 2695 HPLC system with autosampler and Waters 2996 diode array detector.

Column: Macherey-Nagel Nucleoshell RP18 plus (5 µm, 4 mm x 100 mm).

Column temperature: 40°C.

Solvents: A (H_2O 99.9%, H_2CO_2 0.1%); B (CH_3CN 99.9%, H_2CO_2 0.1%).

Flow rate: 1 mL/min.

Gradient (A/B v/v): 90/10 (t = 0 min), 90/10 (t = 1 min), 0/100 (t = 7 min), 0/100 (t = 10 min).

Detection: 210-400 nm range.

Chiral Chromatography:

Chiral column: Daicel ChiralPak IG (Amylose-based) 20 µm, 4.6 mm x 100 mm.

Chiral column: Daicel ChiralPak IG (Amylose-based) 5 µm, 4.6 mm x 250 mm.

Column temperature: 25°C.

Analysis of compound 3 (Isocratic conditions) : Solvents Heptane 95% / EtOH containing 0.1% Et_3N 5%, flow rate: 1mL/min.

Analysis of compound 7 (Isocratic conditions) : Solvents Heptane 90% / EtOH containing 0.1% Et_3N 10%, flow rate: 1mL/min.

Analysis of compound 12 (Isocratic conditions) : Solvents Heptane 41.5% / EtOH containing 0.1% Et_3N 32.5% and DCM 25%, flow rate: 1mL/min.

Analysis of final compound A (Isocratic conditions): Solvents Heptane 50% / EtOH containing 0.1% Et_3N 40% and DCM 10%, flow rate: 1mL/min.

Mass Spectrometer:

Apparatus: Waters Micromass ZQ (simple quad).

Mass detection method: Electrospray positive mode (ESI+), mass range: 50-800 uma.

NMR Spectrometer:

Apparatus: Bruker 400 MHz.

Methods: ^{1}H NMR spectra performed in DMSO-d6 using DMSO-d5 as internal reference, chemical shifts expressed in parts per million (ppm),

^{31}P NMR spectra performed in DMSO-d6, chemical shifts expressed in parts per million (ppm)
